# Artificial Intelligence Predicted Overall Survival and Classified Mature B-Cell Neoplasms Based on Immuno-Oncology and Immune Checkpoint Panels

**DOI:** 10.3390/cancers14215318

**Published:** 2022-10-28

**Authors:** Joaquim Carreras, Giovanna Roncador, Rifat Hamoudi

**Affiliations:** 1 Department of Pathology, School of Medicine, Tokai University, 143 Shimokasuya, Isehara 259-1193, Kanagawa, Japan; 2 Monoclonal Antibodies Unit, Spanish National Cancer Research Center (Centro Nacional de Investigaciones Oncologicas, CNIO), Melchor Fernandez Almagro 3, 28029 Madrid, Spain; 3 Department of Clinical Sciences, College of Medicine, University of Sharjah, Sharjah P.O. Box 27272, United Arab Emirates; 4 Division of Surgery and Interventional Science, University College London, Gower Street, London WC1E 6BT, UK

**Keywords:** non-Hodgkin lymphoma, mature B-cell neoplasms, immune checkpoint, immuno-oncology, immune microenvironment, 3D macrophages, artificial intelligence, machine learning, artificial neural networks, deep learning

## Abstract

**Simple Summary:**

Artificial intelligence (AI) is a field that combines computer science with robust datasets to solve problems. AI in medicine uses machine learning and deep learning to analyze medical data and gain insight into the pathogenesis of diseases. This study summarizes and integrates our previous research and advances the analyses of macrophages. We used artificial neural networks and several types of machine learning to analyze the gene expression and protein levels by immunohistochemistry of several hematological neoplasia and pan-cancer series. As a result, the patients’ survival and disease subtype classification were achieved with high accuracy. Additionally, a review of the literature on the latest progress made by AI in the hematopathology field and future perspectives are given.

**Abstract:**

Artificial intelligence (AI) can identify actionable oncology biomarkers. This research integrates our previous analyses of non-Hodgkin lymphoma. We used gene expression and immunohistochemical data, focusing on the immune checkpoint, and added a new analysis of macrophages, including 3D rendering. The AI comprised machine learning (C5, Bayesian network, C&R, CHAID, discriminant analysis, KNN, logistic regression, LSVM, Quest, random forest, random trees, SVM, tree-AS, and XGBoost linear and tree) and artificial neural networks (multilayer perceptron and radial basis function). The series included chronic lymphocytic leukemia, mantle cell lymphoma, follicular lymphoma, Burkitt, diffuse large B-cell lymphoma, marginal zone lymphoma, and multiple myeloma, as well as acute myeloid leukemia and pan-cancer series. AI classified lymphoma subtypes and predicted overall survival accurately. Oncogenes and tumor suppressor genes were highlighted (MYC, BCL2, and TP53), along with immune microenvironment markers of tumor-associated macrophages (M2-like TAMs), T-cells and regulatory T lymphocytes (Tregs) (CD68, CD163, MARCO, CSF1R, CSF1, PD-L1/CD274, SIRPA, CD85A/LILRB3, CD47, IL10, TNFRSF14/HVEM, TNFAIP8, IKAROS, STAT3, NFKB, MAPK, PD-1/PDCD1, BTLA, and FOXP3), apoptosis (BCL2, CASP3, CASP8, PARP, and pathway-related MDM2, E2F1, CDK6, MYB, and LMO2), and metabolism (ENO3, GGA3). In conclusion, AI with immuno-oncology markers is a powerful predictive tool. Additionally, a review of recent literature was made.

## 1. Introduction

Lymphoid neoplasms are tumors of the hematopoietic system derived from immature and mature B lymphocytes, T lymphocytes, and natural killer (NK) cells that evoke the normal stages of cell differentiation. Nevertheless, some neoplasms (such as hairy cell leukemia) show lineage heterogeneity and plasticity, and their normal counterparts cannot be found [1,2,3,4,5,6,7]. The 2016 revision of the World Health Organization (WHO) classification of lymphoid neoplasms [3] and the International Consensus Classification (ICC) [6] describe around 45 different subtypes of mature lymphoid neoplasms [3,6,7]. In this research, we analyzed the gene expression of some of the most relevant and frequent ones.

Chronic lymphocytic leukemia/small lymphocytic lymphoma (CLL/SLL) develops from small mature CD5+ and CD23+ B-cells with mutated or unmutated *IGHV* genes [3,8].

Follicular lymphoma (FL) is a neoplasia of the germinal centers of follicles (centrocytes and centroblasts), with a follicular (nodular) pattern, and is frequently associated with the *IGH*/*BCL2* translocation (t14;18)(q32;q21) that occurs in the bone marrow [3,9,10].

Extranodal marginal zone lymphoma of mucosa-associated lymphoid tissue is an extranodal lymphoma (MALT lymphoma) composed of a heterogeneous population of small B-cells [3]. It originates in the marginal zones, but it extends into the interfollicular and follicular regions and infiltrates the epithelium, forming the lymphoepithelial lesions [3,11].

Mantle cell lymphoma (MCL) is characterized by monomorphic small to medium-sized lymphoid cells with irregular nuclei and the *CCND1* translocation, originating from peripheral B lymphocytes of the inner mantle zone, CD5+, and SOX11+ in the classical form [3,12,13].

Diffuse large B-cell lymphoma (DLBCL) is a neoplasm of medium or large B lymphoid cells that originate from the germinal center in the germinal center B-cell-like type, or from the post-germinal center in the activated B-cell-like type [3,14,15]. According to the clinical, morphological, and biological features, DLBCL can be subdivided into different subtypes; the remaining ones are not otherwise specified (NOS).

Burkitt lymphoma is a highly aggressive but curable lymphoma that often appears at extranodal sites or as acute leukemia. It is characterized by a monomorphic proliferation of medium-size B-cells, mitotic figures, and the *MYC* translocation to the immunoglobulin (IG) locus. It originates from the germinal centers. There are three epidemiological variants, with variable association with the Epstein-Barr virus (EBV): endemic, sporadic, and immunodeficiency-associated [3,16,17,18].

**Figure 1:**
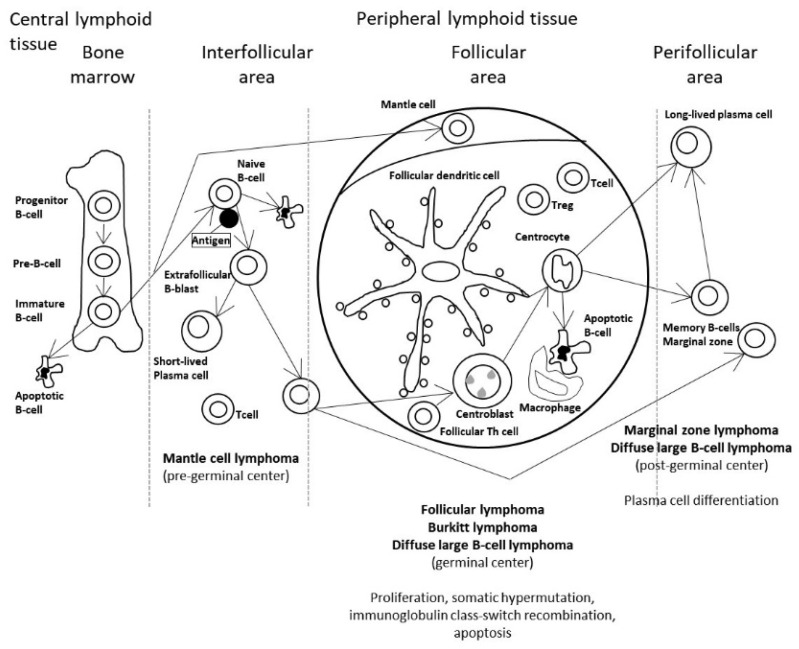
Postulated cell of origin of the non-Hodgkin lymphoma subtypes. In the current theory of the pathogenesis of hematopoietic and lymphoid tissues, B-cell neoplasms correspond to various stages of B-cell differentiation. For example, follicular lymphoma, Burkitt lymphoma, and diffuse large B-cell lymphoma develop (or have a stage of differentiation) from mature B lymphocytes from the germinal centers of follicles of peripheral lymphoid tissues. Of note, follicular lymphoma is characterized by the IGH/BCL2 translocation (t14;18)(q32;q21) that occurs in the bone marrow. Nevertheless, this genetic alteration is not sufficient to generate lymphoma, and additional cumulative changes are necessary.

Figure 1 shows the stages of the B-lymphocyte differentiation, and the relationship with the different lymphoma subtypes [19].

Nowadays, there has been rapid advance in the field of artificial intelligence (AI), and its role in medicine is gaining relevance. AI integrates computer science and datasets to make predictions or classifications based on input data.

There are two types of artificial intelligence, weak and strong AI. Weak AI, also known as narrow AI (NAI), is trained to perform specific tasks. Conversely, strong AI includes artificial general intelligence (AGI) or artificial super intelligence (ASI), and it is expected to surpass human abilities in the future [20,21,22,23,24,25,26].

**Figure 2:**
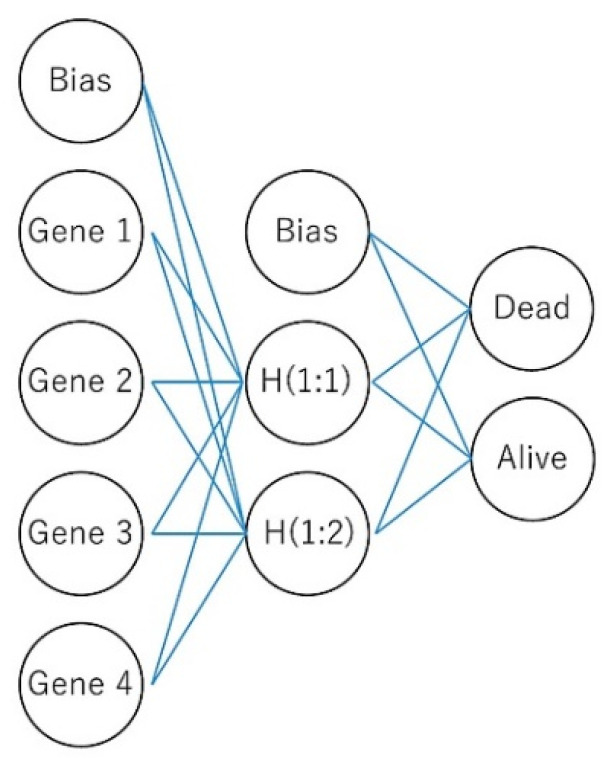
The basic structure of a neural network. The network is a function of predictors (also called inputs or independent variables) that minimize the prediction error of target variables (outputs). In the case of a multilayer perceptron, it is a feed-forward architecture because the connections flow from the input to the output layer without loops. Here, four genes predict the overall survival of patients. The input layer contains these genes. The hidden layer contains the unobservable nodes (units). The output layer contains the responses; the overall survival is a categorical variable (dead vs alive).

In this research, we used weak artificial intelligence to predict the prognosis of the patients and to classify several subtypes of mature B-cell neoplasms (output). Gene expression (transcriptomics) and protein immunohistochemical data were used as predictors (input data). The research focused on artificial neural networks (mainly multilayer perceptron), but also used other neural networks such as the radial basis function and other machine learning techniques. Regarding the neural networks, “basic” but robust and reliable architectures were chosen as an elemental part of the analysis. Then, the “basic” networks were combined in more complex, multivariate analysis algorithms. Figure 2 describes the basic structure of the neural network.

The immune checkpoints are regulators of the immune system that belong to the self-tolerance pathways. Without them, the immune system would attach to cells indiscriminately. Cancer uses several mechanisms to proliferate, including evading the host immune response using immune checkpoint molecules. There are two types of immune checkpoint molecules: stimulatory and inhibitory. Inhibitory checkpoint molecules inhibit the immune response and include several markers such as B7-H3 (CD276), BTLA, CTLA-4, LAG3, PD-1, TIM-3, and VISTA. Nowadays, immune checkpoints are important because they are the basis of cancer immunotherapy. Currently approved checkpoint inhibitors are anti CTLA-4, PD-1, and PD-L1 [19,27,28,29,30,31,32,33,34,35]. In this research, artificial intelligence was used to classify and to predict the overall survival of different lymphoma subtypes using gene expression data, all the genes of the arrays, and specific panels of the immune checkpoint.

This manuscript integrates our previous publications to provide a general view of the results and adds new analysis on tumor-associated macrophages (TAMs).

## 2. Materials and Methods

### 2.1. Machine Learning and Neural Networks

This research integrates all the previous analyses that were obtained using conventional biostatistics, machine learning, and artificial neural networks. Machine learning included Bayesian network, C&R tree, C5 tree, CHAID tree, discriminant analysis, KNN algorithm, logistic regression, LSVM, Quest tree, random forest, random trees, SVM, tree-AS, XGBoost linear, and XGBoost tree. Two types of artificial neural networks were used: the multilayer perceptron and radial basis function. The digital image quantification of markers was performed using the Waikato Environment for Knowledge Analysis (Weka), and the training of the classifier included fast random forest. All the materials and methods were thoroughly described in the previous publications [19,27,28,29,30,31,32,33,34,35].

### 2.2. Multilayer Perceptron Artificial Neural Network

The multilayer perceptron architecture was chosen in most cases. Several parameters were chosen to optimize the neural network. The predictors were included in the input layer, the unobservable nodes or units in the hidden layer, and the responses in the output layer. Scale-dependent variables and covariates were rescaled to improve network training. The method for rescaling of covariates was standardized: subtract the mean and divide by the standard deviation, (*x*−mean)/*s*.

The series of cases were randomly partitioned into training (70%) and testing (30%) datasets. The best performance was found using one hidden layer. The activation function linked the weighted sums of units in a layer to the values of units in the succeeding layer. The hyperbolic tangent was usually used. This function has the form γ(*c*) = tanh(*c*) = (*e^c^ –e^–c^*)/(*e^c^ +e^−c^*). It takes real-valued arguments and transforms them into the range (–1, 1). When automatic architecture selection is used, this is the activation function for all units in the hidden layers. The number of units in each hidden layer was determined automatically by an estimation algorithm.

The output layer contained the target (dependent) variables and the activation function was softmax. This function has the form: γ(*c*_k_) = exp(*c*_k_)/Σ_j_exp(*c*_j_). It takes a vector of real-valued arguments and transforms it into a vector whose elements fall in the range (0,1) and sum to 1. Softmax is available only if all dependent variables are categorical.

**Figure 3:**
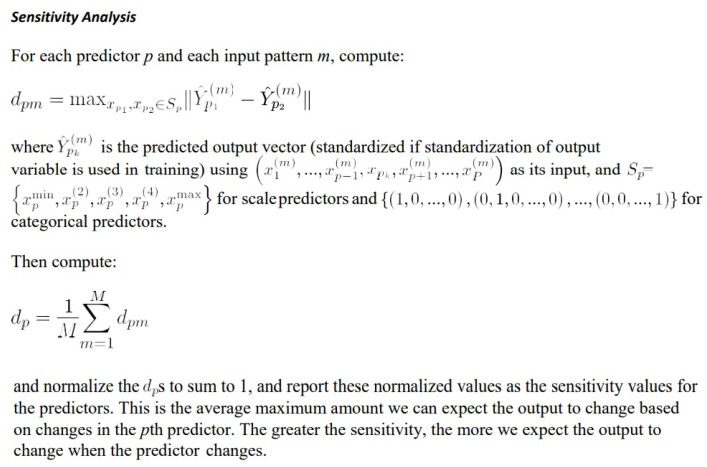
Sensitivity analysis. Using a sensitivity analysis, the independent variables were ranked according to their importance for predicting the dependent variable and in determining the neural network.

The training type determines how the network processes the records; the training type was batch. The training options were initial lambda (0.0000005), initial sigma (0.00005), interval center (0), and interval offset (+/−0.5). The network performance was assessed by the classification results, receiver operating characteristic (ROC) curve, cumulative gains chart, lift chart, predicted by observed chart, and residual by predicted chart. Using a sensitivity analysis, the independent variables were ranked according to their importance for predicting the dependent variable and in determining the neural network (Figure 3).

The general architecture for a multilayer perceptron is as follows [34]:

Input layer: *J*_0_ = *P* units, *a*_0:1_, …, *a*_0:*J*0_; with *a*_0:*j*_ = *x_j_*.

Hidden layer: *J*_i_ units, *a_i_*_:1_, …, *a_i:Ji_*; with *a_i:k_* = γ*_i_*(*c_i:k_*) and *c_i:k_* = $\sum_{j=0}^{\mathrm{Ji}-1}$ *w_i:j,k_a_i_*__1:*j*_ where

.*a_i_*_−1:0_ = 1

Output layer: *J*_I_ = *R* units, *a_I_*_:1_, …, *a_I:Ji_*; with *a_I:k_* = γ*_I_*(*c_I:k_*) and *c_I:k_* = $\sum_{j=0}^{J1}$ *w_I_*_:*j,k*_*a*_i_ 1:*j*_ where .*a_i_*_−1:0_ = 1

Notation [34]:

*I*  Number of layers, discounting the input layer.
*J_i_*  Number of units in layer i. *J*_0_ = *P*,*J*_i_ = *R*, discounting the bias unit.
*w_i_*_:*j,k*_ Weight leading from layer *i–*1, unit *j* to layer *i*, unit *k*. No weights connect $a_{\text{i}-1:j}^{m}$ and the bias $a_{\text{i}-j:0}^{m}$; that is, there is no *w*_i:*j,*0_ for any *j*.
γ*_i_*(*c*) Activation function for layer *i*.
*w*  Weight vector containing all weights (*w*_1:0,1,_ *w*_1:0,2, …,_ *w_I_*_:*JI*−*1*,*JI*_).

### 2.3. Differential Gene Expression Using the GEOR2 Software

The GEO2R 1.0 software was used to compare the differential gene expression between subtypes simply. The Benjamini–Hochberg false discovery rate was applied to adjust the *p* values. Log transformation was applied if necessary. Limma precision weights and force normalization were not applied. The data were visualized using volcano and mean difference (MA) plots, contrasted with a level of cut-off significance set a priori at 0.05. This software runs in R 3.2.3, Biobase 2.30.0, GEOquery 2.40.0, limma 3.26.8. Webpage: https://www.ncbi.nlm.nih.gov/geo/info/geo2r.html (accessed on 23 July 2022).

### 2.4. Gene Set Enrichment Analysis

The Gene Set Enrichment Analysis (GSEA) was used to determine if a pathway of interest was associated with a particular biological state (for example, dead vs alive) [36,37]. The pathways were obtained from the Molecular Signatures Database (MSigDB 7.0 and greater) or designed in-house. The software GSEA v4.2.3 was downloaded from the webpage of UC San Diego, Broad Institute: http://www.gsea-msigdb.org/gsea/index.jsp (accessed on 23 July 2022).

### 2.5. Conventional Statistical Analyses

Comparisons between groups were performed using crosstabulation with Pearson Chi-Square and Fisher’s exact tests, and with nonparametric Mann–Whitney U (2 groups) and Kruskal-Wallis H (≥3 groups) tests. Survival analyses used the Kaplan–Meier and Log-rank tests, and the univariate and multivariate Cox Regression. The criteria of survival and response were the standard [38]. Overall survival was calculated from the time of diagnosis to the last contact with the patient (event recorded as alive vs dead).

### 2.6. Risk Groups

Risk groups were created using the risk score (prognostic index), which was calculated by multiplying the beta coefficients of the Cox model by the gene expression values (Risk score = B_1_X_1_ + B_2_X_2_ + … + B_p_X_p_, where x_i_ is the expression value and B_I_ is the beta value of the Cox table). In the Cox, all the genes are included in a unique model [39].

### 2.7. Hardware

The analyses were performed on a desktop equipped with an AMD Ryzen 5 1600 and NVIDIA GeForce GTX 1050 Ti [27], Ryzen 7 3700X and GeForce GTX 1650 [30,33,34], and a Ryzen 9 5900X and GeForce GTX 3060 Ti [35], all with 16.0 GB of RAM.

Appendix A describes all the software that was used to perform the biostatistical analyses, including machine learning and artificial neural networks [19,27,28,29,30,31,32,33,34,35].

### 2.8. Datasets and Immunohistochemical Procedures

We used publicly available datasets downloaded from the Gene Expression Omnibus (GEO) repository, webpage: https://www.ncbi.nlm.nih.gov/geo/ (accessed on 23 July 2022) (Appendix B Table A1) [40,41,42,43,44,45,46,47,48,49,50,51,52,53,54,55,56,57], and own Tokai University Hospital gene expression (transcriptomic) and immunohistochemical (proteomic) datasets for this research.

Several of the markers that were highlighted in the AI analyses (both machine learning and artificial neural network) were validated by immunohistochemistry at the protein level. The cases were selected from the lymphoma series of Tokai University Hospital. The series of cases ranged from 100 to 293 cases, depending on the project. Immunohistochemistry was performed using a Leica Bond Max autostainer following the manufacturer’s instructions (Leica K.K., Tokyo, Japan). Table 1 details the primary antibodies that were used. The review section was made on the basis of PRISMA guidelines: https://prisma-statement.org/ (accessed on 29 September 2022), Carreras, J. (20 October 2022). Systematic review. https://doi.org/10.17605/OSF.IO/436JQ. The manuscripts were selected in PubMed using the keywords “lymphoma” and “artificial intelligence”, and were organized according to the type of input data as PET/CT scan, histological images, immunophenotype, clinicopathological variables, and gene expression, mutational, and integrative analysis-based artificial intelligence.

## 3. Results

The different subtypes of hematological neoplasia (mainly non-Hodgkin lymphomas) were predicted using artificial neural networks, machine learning, and conventional biostatistics. The analysis used transcriptomic data and protein levels assessed by immunohistochemistry. The results are summarized as a bulleted list.

### 3.1. Predictive Classification of Non-Hodgkin Lymphomas

**Figure 4:**
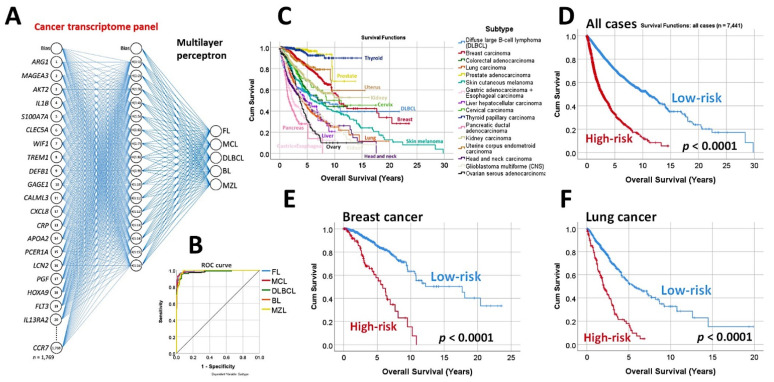
Prediction of lymphoma subtype by a neural network with high accuracy. (**A**) A multilayer perceptron predicted the different non-Hodgkin lymphoma subtypes, including follicular lymphoma, mantle cell lymphoma, diffuse large B-cell lymphoma, Burkitt’s lymphoma, and marginal zone lymphoma. The predictors (inputs) were the gene expression values of a pan-cancer transcriptome panel. The architecture of the network had 1769 nodes in the input layer, a hidden layer of 16 nodes, and an output layer with 5 nodes (5 lymphoma subtypes). In this figure, the top 20 most relevant genes for predicting the lymphoma subtype are shown, based on their average normalized importance for prediction. The most relevant gene was *ARG1*, followed by *MAGEA3*, *AKT2*, and *IL1B*. (**B**) This multilayer perceptron had a high performance, as shown in the ROC curve that had an area under the curve near 1. (**C**–**F**) Interestingly, the top 30 genes of the neural network not only predicted the lymphoma subtype but also managed to predict the overall survival of a large pan-cancer series from the TCGA of 7441 cases. Using a risk score formula, the cases of each series were stratified into high- and low-risk groups. The risk scores were calculated by multiplying the beta values of the Cox regression per gene expression values for each gene. The overall survival was calculated using the Kaplan–Meier and log-rank test and Cox regression analyses. These top 30 genes belonged to a pan-cancer transcriptome panel. Therefore, this may explain why they have predictive value in a pan-cancer series, and points out that there may be common cancer mechanisms in all human neoplasia.

- Using the whole array of 20,863 and a cancer transcriptome panel, the lymphoma subtypes were predicted by a neural network with high accuracy [19].
- A set of 30 genes derived from the neural network also predicted the overall survival of an independent series of diffuse large B-cell lymphoma, and a pan-cancer series of 7441 cases of The Cancer Genome Atlas (TCGA) [19] (Figure 4).

### 3.2. Follicular Lymphoma, Immune Response, and Microenvironment

**Figure 5:**
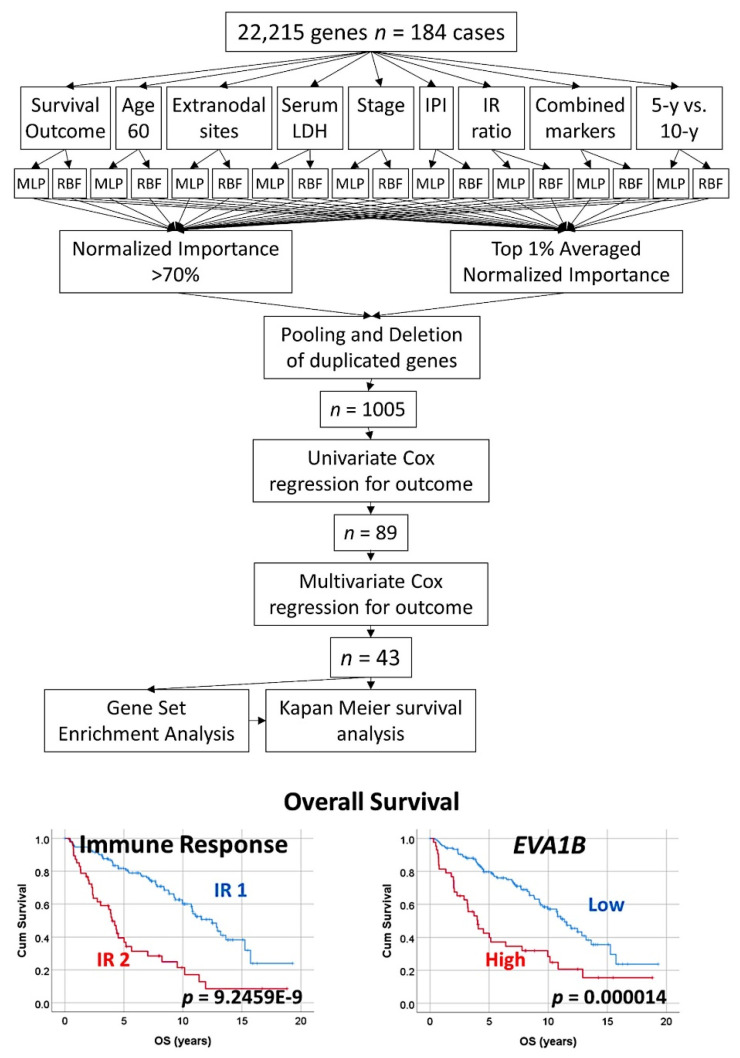
Prediction of the overall survival of follicular lymphoma using an algorithm based on neural networks. The algorithm combined multilayer perceptron (MLP), radial basis function (RBF), and COX regression to highlight 43 genes with prognostic relevance; finally, a correlation with immuno-oncology genes was also performed. This figure shows the algorithm (method) that was used to analyze the gene expression data of follicular lymphoma using artificial neural networks. From an initial set of 22,215 genes, a strategy of dimensionality reduction highlighted 43 genes, of which 18 were associated with poor and 25 with good overall survival of the patients. The first step consisted of several independent artificial neural networks. The network architecture included the 22,215 genes as predictors (inputs), a hidden layer, and an output layer with the predicted variable. The predicted variables were the overall survival of the patients (outcome dead vs alive), and other relevant clinicopathological variables of follicular lymphoma. The result of the neural network ranked all the genes according to their normalized importance for predicting the target variable. The results of the independent multiple neural networks were pooled resulting in 1005 genes, and the most relevant ones were highlighted using univariate and multivariate Cox regression analyses. The relevance of these genes was confirmed using gene set enrichment analysis (GSEA). Finally, these genes were also correlated with several immuno-oncology genes. The 43 genes were the following: 18 were associated with a poor prognosis (*FRYL, KIAA0100, CDC40, MED8, PTP4A2, BNIP2, TMEM70, MED6, SLC24A2, KLK10, RANBP9, PRB1, EVA1B, CBFA2T2, ALDH1L1, KRT19, BTN2A3P,* and *TRPM4*) and 25 were associated with a good prognosis of the patients (*HSF2, ATPAF2, SLC7A11, PTAFR, TTLL3, TCP10L, DNAAF1, PRH1, NSDHL, TAF12, TSPAN3, AKIRIN1, ITK, TDRD12, LPP, BTD, SIRT5, ZNF230, ABHD6, TOP2B, ARPC2, ASAP2, IDH3A, PSMF1,* and *ARFGEF1*) (Supplementary Tables S1–S5). LDH, lactate dehydrogenase; IPI, international prognostic index; IR ratio, immune response ratio; 5-y, five years; MLP, multilayer perceptron; RBF, radial basis function.

**Figure 6:**
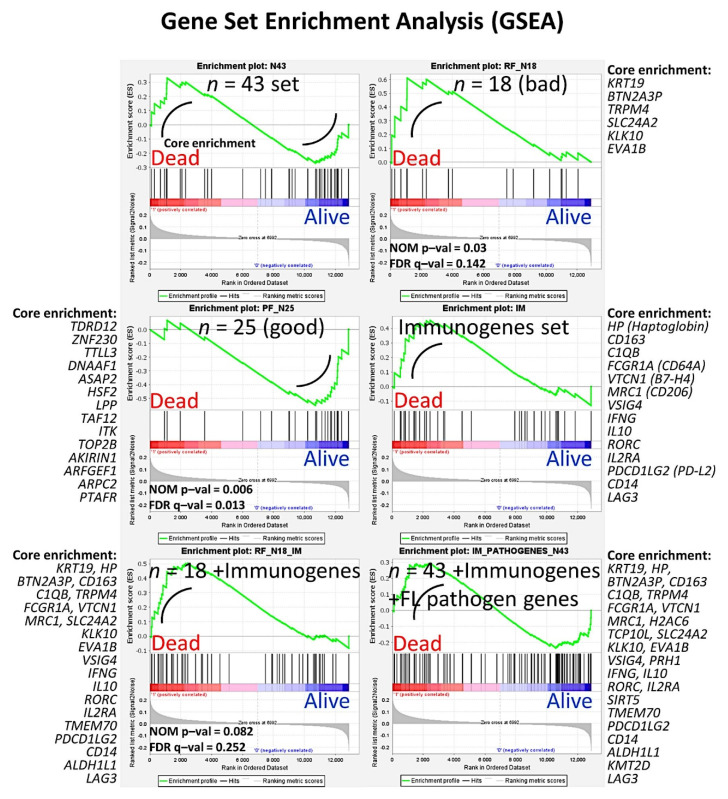
Prediction of the overall survival of follicular lymphoma using an algorithm based on neural networks. This figure shows the GSEA results of Figure 4 in detail. Gene set enrichment analysis (GSEA) was performed to confirm the results of the multivariate Cox regression for the overall survival analysis. The set of 43 was used in addition to genes of the immune response as well as oncogenes and tumor suppressor genes related to the pathogenesis of follicular lymphoma. Of note, genes related to macrophages were highlighted, such as *CD163*. NOM p–val, nominal p value (the nominal *p* value estimates the statistical significance of the enrichment score for a single gene set); FDR q–val, false discovery rate.

**Figure 7:**
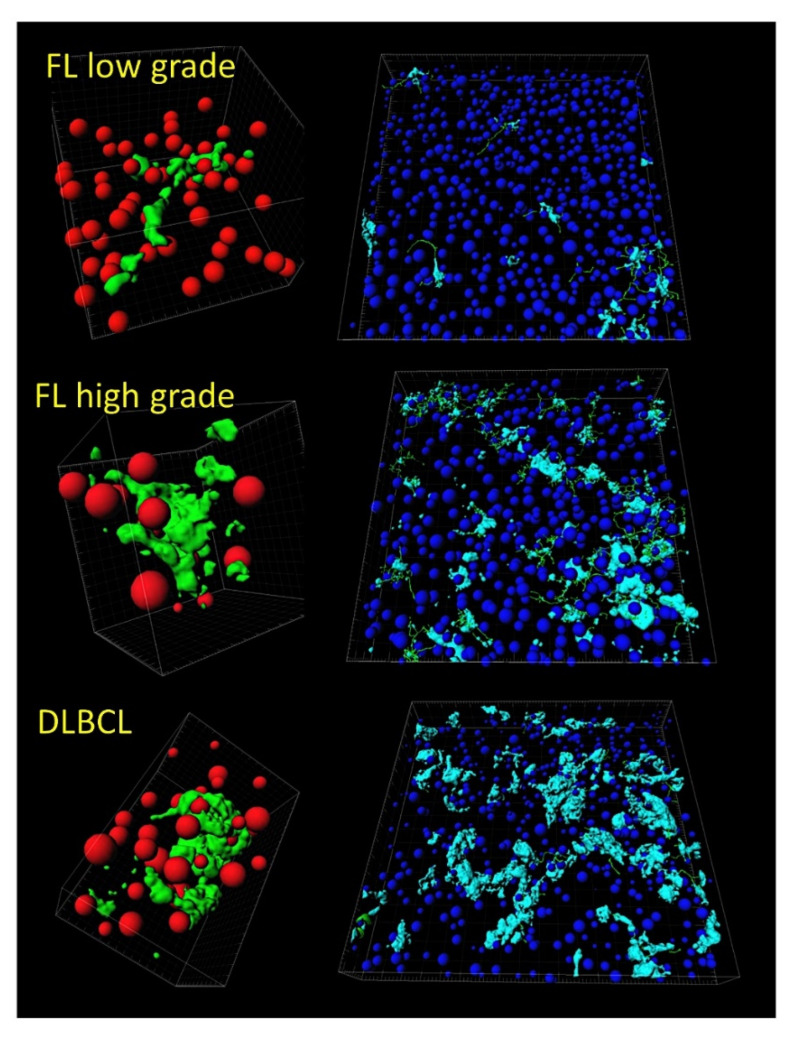
Tridimensional analysis of tumor-associated macrophages (TAMs) in follicular lymphoma. The analysis of M2-like TAMs in follicular lymphoma showed that the progression from low grade to high grade, and the transformation to diffuse large B-cell lymphoma, were associated with increased numbers of TAMs, which created a physical network-like structure. This result points out that TAMs may contribute to the disease pathogenesis. In this figure, the macrophages are highlighted in pale blue (right) and green (left). B and T lymphocytes are in dark blue and red. The images were obtained using a LSM 700 laser scanning confocal microscope from Carl Zeiss (Carl-Zeiss-Strasse 22, 73447 Oberkochen, Germany), and Imaris software (version 8.4, Oxford Instruments, Belfast, United Kingdom). FL, follicular lymphoma; DLBCL, diffuse large B-cell lymphoma.

- An algorithm combined two types of neural networks (multilayer perceptron and radial basis function) to predict the overall survival, in combination with other clinically relevant variables [29].
- These variables were more than 60 years, the number of extranodal sites > 1, LDH-level ratio > 1, stage > 2, IPI score 2−3, with translocation (14;18) positive, immune response ratio 2:1 high (≥0.97), and overall survival up to 5 years vs alive from 10 years [29].
- As a result, new poor and favorable prognostic genes were identified, and were correlated with the immune microenvironment (M2-like tumor-associated macrophages) [29] (Figure 5 and Figure 6).
- Tridimensional (3D) analysis of tumor-associated macrophages (TAMs) of follicular lymphoma and transformation to diffuse large B-cell lymphoma was associated with increased numbers of TAMs, which created a network-like structure (Figure 7).

### 3.3. Follicular Lymphoma, Random Number Generator-Based Strategy

**Figure 8:**
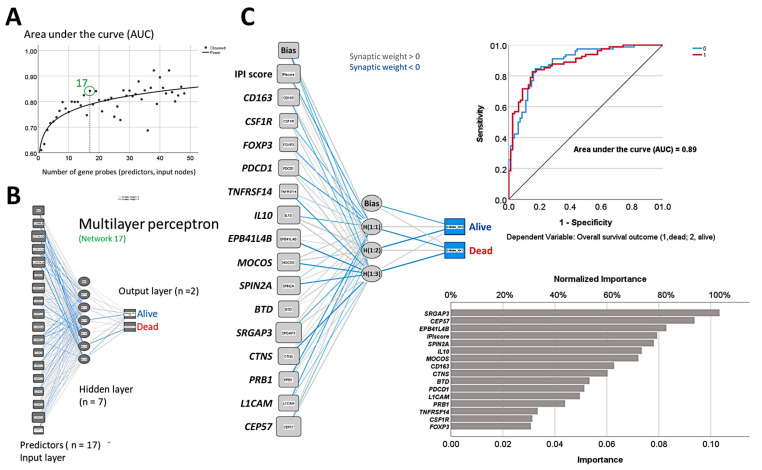
Prediction of the overall survival of follicular lymphoma taking advantage of the random number generator. (**A**) By using the random generator, 120 independent and different neural network solutions were calculated, and the averaged normalized importance of each gene for predicting the overall survival was recorded. Then, the minimal number of genes of a neural network with sufficient performance was selected, and a final neural network with 17 genes was defined. (**B**) This neural network (multilayer perceptron type) included 17 genes in the input layer, a hidden layer of 7 nodes, and an output layer of 2 nodes (overall survival, death vs alive). (**C**) A new neural network was created with the highlighted 17 genes and known immuno-oncology genes. The resulting model had an acceptable accuracy, with an area under the curve (AUC) of 0.89. The predictors (inputs) were ranked according to their normalized importance in predicting the overall survival.

- The random number generation created 120 independent multilayer perceptron solutions and 22,215 gene probes were ranked according to their averaged normalized importance for predicting the overall survival [35].
- The analysis identified new predictor genes, which were related to cell adhesion and migration, cell signaling, and metabolism. These genes were also correlated to the immuno-oncology markers of *CD163, CSF1R, FOXP3, PDCD1* *(PD-1), TNFRSF14 (HVEM)*, and *IL10* [35].
- A comparison with other machine learning techniques was also performed. Machine learning included the following techniques: Bayesian network, C&R tree, C5 tree, CHAID tree, discriminant analysis, KNN algorithms, logistic regression, LSVM, Quest tree, random forest, random trees, SVM, tree-AS, XGBoost linear, and XGBoost tree. A neural network analysis was also made [35] (Figure 8).

### 3.4. Mantle Cell Lymphoma, Use of Immuno-Oncology Panels to Predict Survival

**Figure 9:**
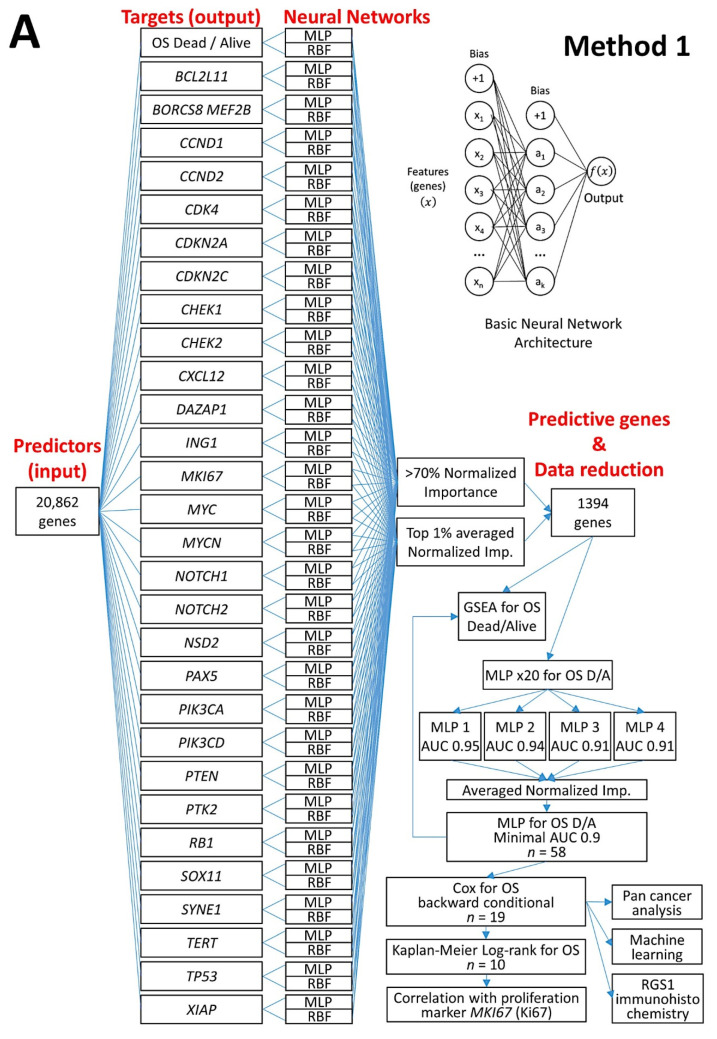

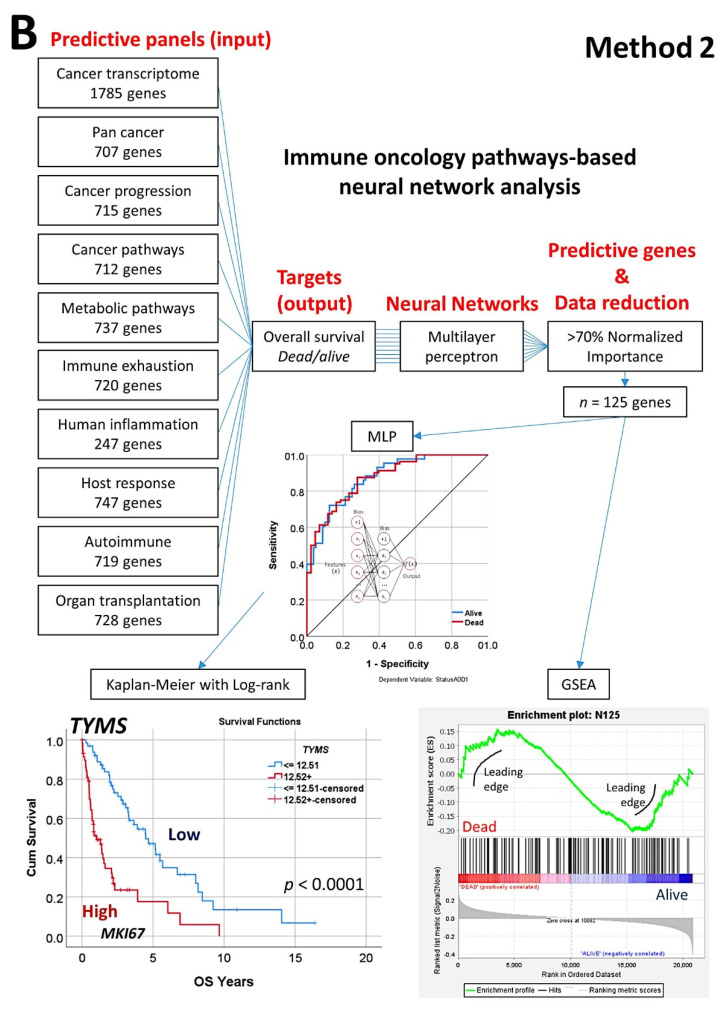
Prediction of the overall survival of mantle cell lymphoma using an algorithm based on neural networks. Two methods (**A** and **B** algorithms) were designed. Method 1 used as input 20,862 genes to predict the overall survival outcome (dead vs. alive) and other prognostic markers; because of dimensionality reduction, a final set of 19 genes were highlighted. The analysis also included testing the final 19 genes with other machine learning analysis, and conventional overall survival with log-rank test. Method 2 used as input several gene panels to predict the overall survival. As a result, 125 pan-cancer and immuno-oncology genes were highlighted. The association with the patients overall survival was confirmed by GSEA and conventional overall survival with log-rank test. OS, overall survival; MLP, multilayer perceptron; RBF, radial basis function; GSEA, gene set enrichment analysis; D/A, dead/Alive; AUC, area under the curve; NI, normalized importance.

- An analysis algorithm included several analysis techniques such as neural networks (both the multilayer perceptron artificial and radial basis function), GSEA, and conventional statistics. In this analysis, 20,862 genes were correlated with 28 prognostic genes of mantle cell lymphoma. After dimensionality reduction, the patients’ overall survival was predicted, and new markers were highlighted (Figure 9) [34].
- The highlighted genes were related to the cell cycle, apoptosis, and metabolism. The genes not only predicted the survival of mantle cell lymphoma, but also of diffuse large B-cell lymphoma and a large pan-cancer series of the TCGA [34].
- A neural network algorithm that combined 10 oncology and immuno-oncology panels predicted overall survival (Figure 9) [34].
- Other machine learning techniques were used. Additionally, a correlation with the MCL35 proliferation assay, which was created by the Lymphoma/Leukemia Molecular Profiling Project, was made [34] (Figure 9).

### 3.5. Diffuse Large B-Cell Lymphoma, Identification of the 25 Genes Set

**Figure 10:**
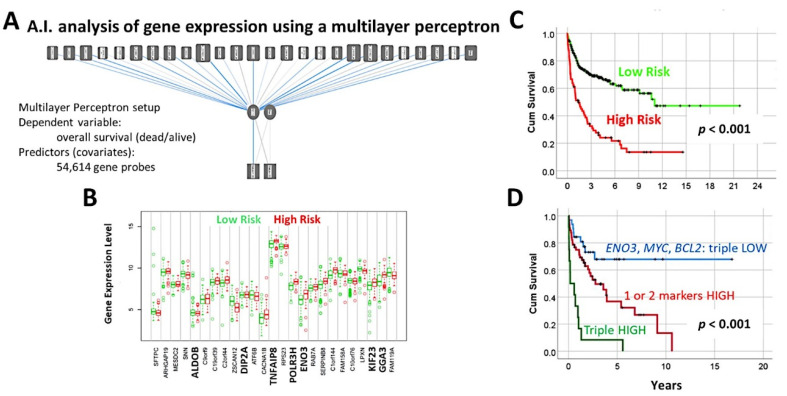
A neural network predicted the overall survival of diffuse large B-cell lymphoma using gene expression data. (**A**) A multilayer perceptron predicted the overall survival and highlighted the most important 25 genes. (**B**) Using a risk score formula and the gene expression of the 25 genes, two groups of patients with different overall survival were found; this figure shows the different gene expression of the 25 genes between the two risk groups. (**C**) The two risk groups had different overall survival. (**D**) Among the 25 genes, *ENO3*, *MYC*, and *BCL2* were the most important, and only with these 3 genes the survival of the patients could be determined.

**Figure 11:**
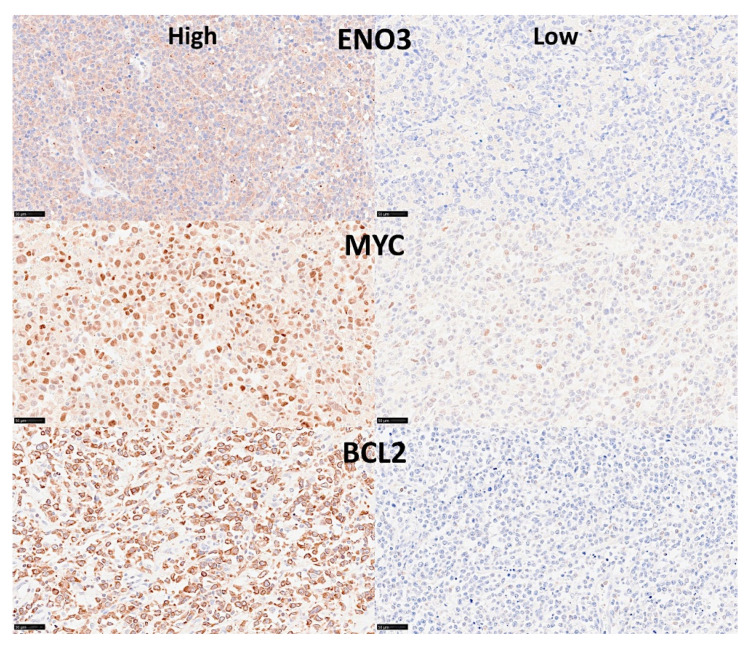
Immunohistochemical staining of ENO3, MYC, and BCL2 in diffuse large B-cell lymphoma. This figure shows six different lymphoma cases, with high or low expression of the 3 markers. Original magnification: 400× (scale bar = 50 um).

- A multilayer perceptron analysis predicted the overall survival of 100 cases using as input 54,614 gene probes, and highlighted 25 genes with prognostic value [27].
- Correlation with known diffuse large B-cell lymphoma markers showed that high expression of MYC, BCL2, and ENO3 was associated with worse outcome [27] (Figure 10 and Figure 11).

### 3.6. Diffuse Large B-Cell Lymphoma, Prognostic Value of the 25 Genes in Hematological Neoplasia, and TNFAIP8 Validation

**Figure 12:**
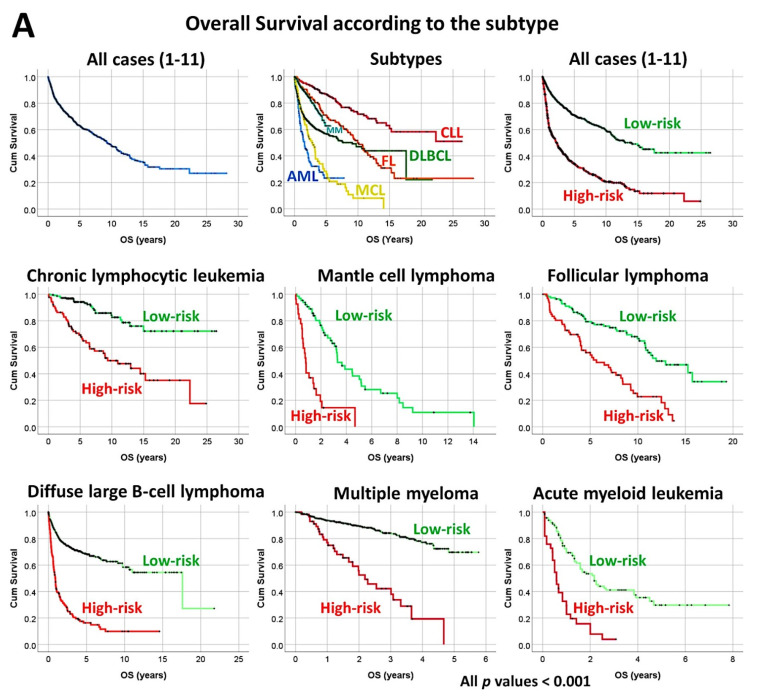

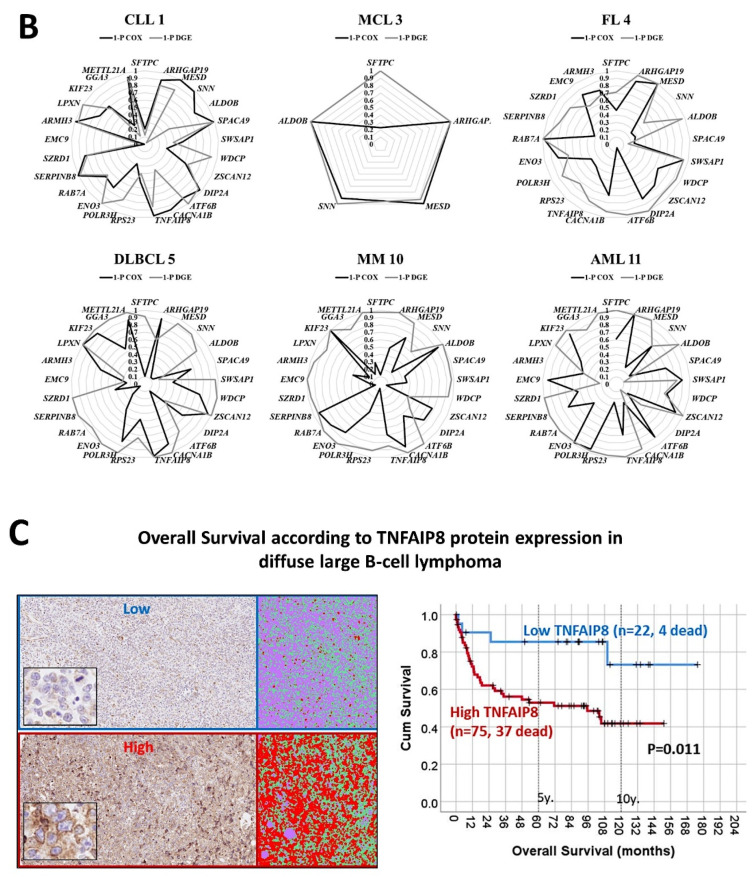
A set of 25 genes derived from a neural network predicted the overall survival of several lymphoma subtypes and acute myeloid leukemia, and high protein expression of TNFAIP8 correlated with poor survival of diffuse large B-cell lymphoma patients. (**A**) Using the gene expression values of 25 genes, previously identified using artificial neural networks, and a risk score formula, it was possible to predict the overall survival of several hematological neoplasia (lymphomas and acute myeloid leukemia). All Kaplan–Meier analyses with log-rank tests were statistically significant and had a *p* < 0.001. (**B**) Although all 25 genes were relevant, the strength and direction of the association was different in each subtype of hematological neoplasia. For example, *TNFAIP8* was more relevant for the overall survival of diffuse large B-cell lymphoma and chronic lymphocytic leukemia, but less relevant for acute myeloid leukemia and multiple myeloma. Nevertheless, *TNFAIP8* contributed to the survival of all these hematological neoplasia. (**C**) High TNFAIP8 protein expression, evaluated by immunohistochemistry using both conventional digital image analysis and AI-based methods, correlated with poor overall survival of diffuse large B-cell lymphoma patients. This figure shows two cases of diffuse large B-cell lymphoma. The figure at the top express low TNFAIP8. On the left, the hematoxylin (dark blue) and DAB-based (brown) immunohistochemical image is shown. As shown in the inset, the TNFAIP8 staining was cytoplasmic. On the right, the AI-based digital image analysis is shown for the same case and area. TNFAIP8 is highlighted in red, cellular structures (B lymphocytes of the lymphoma, T lymphocytes, and macrophages) in pink, and intercellular tissue in green. The figure at the bottom is characterized by high TNFAIP8 expression. After staining procedures, the immunohistochemical slides were digitalized and visualized (NanoZoomer S360 scanner and NDP.view2 viewing software, Hamamatsu KK.). Original magnification: 200×. High TNFAIP8 correlated with age > 60 years, high serum IL2RA, non-GCB phenotype, and high infiltration of CD163+ M2-like tumor-associated macrophages (CD163+TAMs). TNFAIP8 also moderately correlated with MYC (Spearman’s correlation coefficient 0.389, *p* = 0.009) and Ki67 (proliferation index; Spearman’s correlation coefficient 0.48, *p* = 0.001). High TNFAIP8 was also associated (trend) with worse progression-free survival (*p* = 0.052). Finally, a multivariate COX analysis between TNFAIP8 (high vs low) and the international prognostic index (IPI) (low+low/intermediate vs high/intermediate + high) showed that only TNFAIP8 retained the prognostic value (HR = 3.5, *p* = 0.040). CLL, chronic lymphocytic leukemia; DLBCL, diffuse large B-cell lymphoma; FL, follicular lymphoma; MM, multiple myeloma; MCL, mantle cell lymphoma; AML, acute myeloid leukemia.

- The previously identified set of 25 genes not only predicted the prognosis of 741 cases of diffuse large B-cell lymphoma, but also predicted other hematological neoplasia, including chronic lymphocytic leukemia (*n* = 308), mantle cell lymphoma (*n* = 92), follicular lymphoma (*n* = 180), multiple myeloma (*n* = 559), and acute myeloid leukemia (*n* = 149) [28].
- The TNFAIP8 marker was highlighted in this analysis. Because of TNFAIP8’s importance in the apoptotic pathway, it was validated by immunohistochemistry (i.e., at protein level) in an independent series of 97 cases from Tokai University. Digital image quantification of TNFAIP8 was performed using an AI-based method. Correlations with the prognosis of the patients showed that high TNFAIP8 is associated with poor survival [28].
- TNFAIP8 correlated positively with high M2-like CD163-positive tumor-associated macrophages (TAMs) and non-GCB cell of origin phenotype [28] (Figure 12).

### 3.7. Diffuse Large B-Cell Lymphoma, Prediction of Survival by Caspase-8

**Figure 13:**
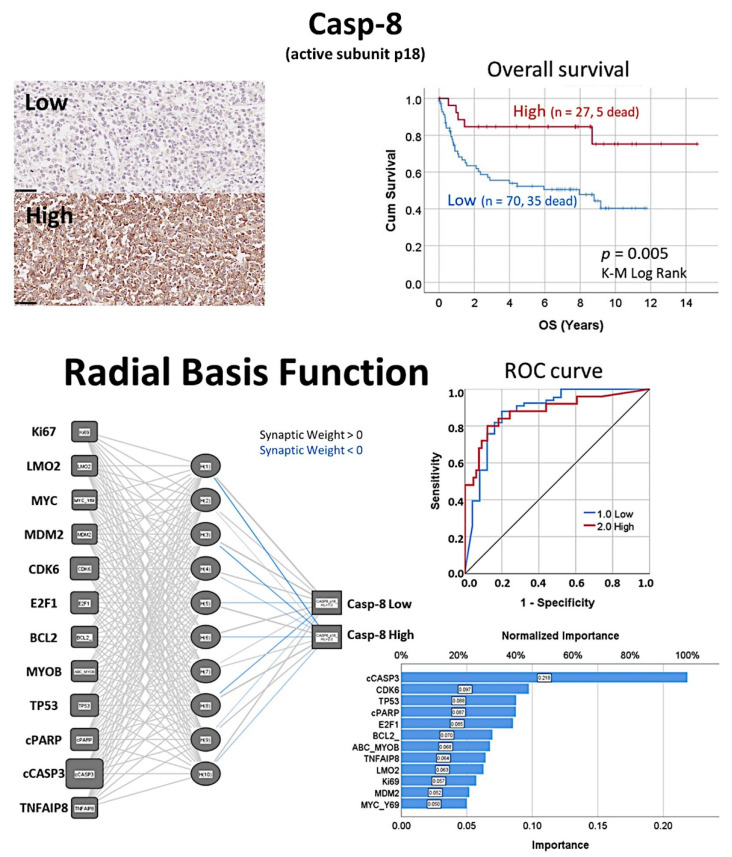
High caspase-8 correlated with favorable survival of diffuse large B-cell lymphoma patients. The protein levels of caspase-8 (*CASP8*) were evaluated by immunohistochemistry, and later correlated with the survival of the patients. Two types of immunohistochemical staining were observed, low and high. In diffuse large B-cell lymphoma, high caspase-8 expression is associated with a favorable overall survival (*p* = 0.005). Additionally, other markers of the capsase-8 pathway, including caspase-3, cleaved PARP, BCL2, TP53, MDM2, MYC, Ki67, E2F1, CDK6, MYB, LMO2, and TNFAIP8, were evaluated by immunohistochemistry and quantified using digital image analysis. Caspase-8 was successfully predicted by the pathway markers, both using conventional statistics and several machine learning techniques and artificial neural networks. Of note, after staining procedures, the immunohistochemical slides were digitalized and visualized (NanoZoomer S360 scanner and NDP.view2 viewing software, Hamamatsu KK.). Original magnification: 400× (scale bar = 50 um). OS, overall survival; ROC curve, the receiver operating characteristic curve.

**Figure 14:**
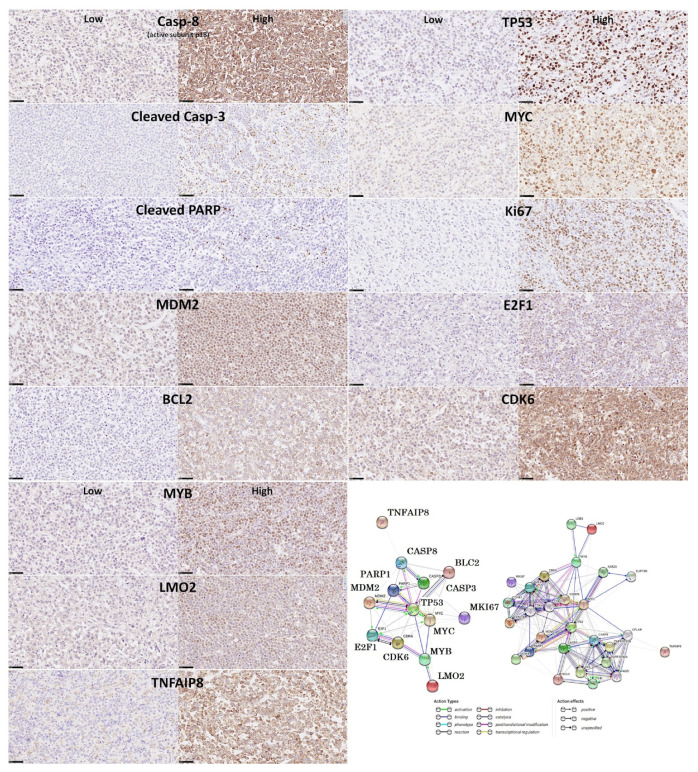
High caspase-8 correlated with favorable survival of diffuse large B-cell lymphoma patients. This figure shows the immunohistochemical expression of active subunit p18 casp-8 (CASP8), which correlated with good prognosis of the patients when high. Other related markers, as shown in the protein–protein interaction analysis, were also analyzed by immunohistochemistry. After staining procedures, the immunohistochemical slides were digitalized and visualized (NanoZoomer S360 scanner and NDP.view2 viewing software, Hamamatsu KK.). All the markers were quantified using digital image analysis. This figure shows examples of low and high expressions for each marker. Original magnification: 400× (scale bar = 50 um).

- The protein expression of caspase-8 (which is inhibited by TNFAIP8) was analyzed by immunohistochemistry in a series of 97 cases of diffuse large B-cell lymphoma, and high expression correlated with a favorable overall and progression-free survival [31].
- Based on an immunohistochemical analysis, caspase-8 was correlated with other markers of its pathway, including BCL2, caspase-3, CDK6, cleaved PARP, E2F1, Ki67, LMO2, MDM2, MYB, MYC, TNFAIP8, and TP53 [31].
- The caspase-8 protein expression was also modeled using several machine learning and artificial neural networks [31] (Figure 13 and Figure 14).

### 3.8. Diffuse Large B-Cell Lymphoma, CD274 (PD-L1) and IKAROS

**Figure 15:**
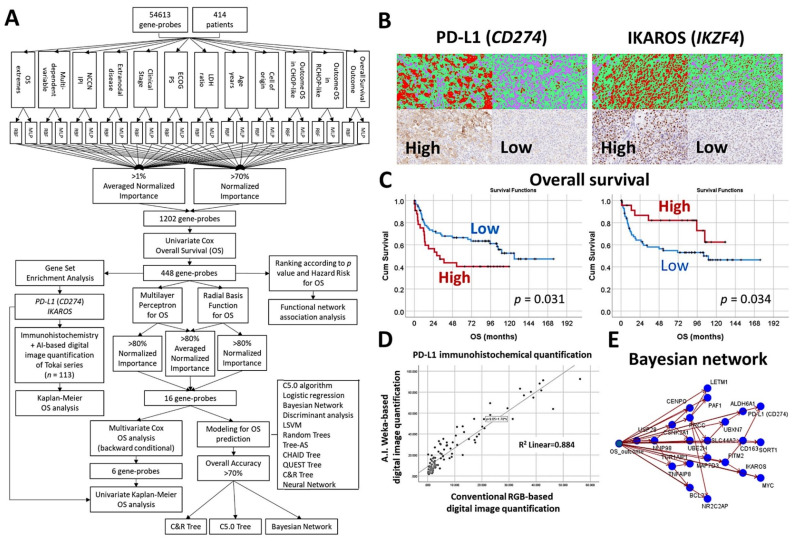
An algorithm that included artificial neural networks and machine learning predicted the survival of diffuse large B-cell lymphoma, and highlighted *PD-L1* and *IKAROS* as prognostic markers. (**A**) Algorithm: This algorithm is similar to that one of follicular lymphoma and mantle cell lymphoma. The basic structure analysis is an artificial neural network (multilayer perceptron). In this analysis, 54,613 gene probes were used as predictors for the overall survival, but also for other relevant clinicopathological variables. The basic neural network was composed of the input layer (predictors, 54,613 gene probes), a hidden layer (automatically computed), and an output layer (predicted variable; for example, the overall survival outcome as a dichotomic variable dead vs alive, or the cell of origin classification (GCB vs ABC), etc.). The dimensionality reduction included additional steps of machine learning, Cox regression, and GSEA. (**B**) Digital image quantification using AI-based strategy for PD-L1 (CD274) and IKAROS. (**C**) High protein expression of PD-L1 correlated with poor survival of the patients. Conversely, high IKAROS was associated with favorable survival. (**D**) AI-based quantification correlated well with conventional digital image quantification. Therefore, both techniques provide comparable results. (**E**) Modeling of the overall survival using a Bayesian network. The Bayesian network builds a probability model, a graphical model that shows variables (nodes) of the dataset, and the probabilistic (conditional) independences between them. The links of the network are called arcs and represent the relationship between the variables, but do not necessarily mean cause and effect. Original magnification: 200×. OS, overall survival; NCCN IPI, National Comprehensive Cancer Network International Prognostic Index; ECOG PS, Eastern Cooperative Oncology Group Performance Status; LDH, lactate dehydrogenase; R-CHOP, rituximab, cyclophosphamide, doxorubicin hydrochloride, vincristine, and prednisolone; AI, artificial intelligence.

- An algorithm included multilayer perceptron, radial basis function, GSEA, COX regression, and several machine learning techniques to predict the overall survival of 414 cases of diffuse large B-cell lymphoma [30].
- The machine learning techniques were Bayesian network, C5.0 algorithm, chi-squared automatic interaction detection CHAID tree, classification and regression (C&R) tree, discriminant analysis, logistic regression, Quest tree, random trees, and tree-AS. The neural network was the multilayer perceptron [30].
- The association of PD-L1 (CD274) and IKAROS with the overall survival was validated in an independent series of 113 cases by immunohistochemistry. The quantification included an AI-based method [30] (Figure 15).

### 3.9. Diffuse Large B-Cell Lymphoma, CSF1R

**Figure 16:**
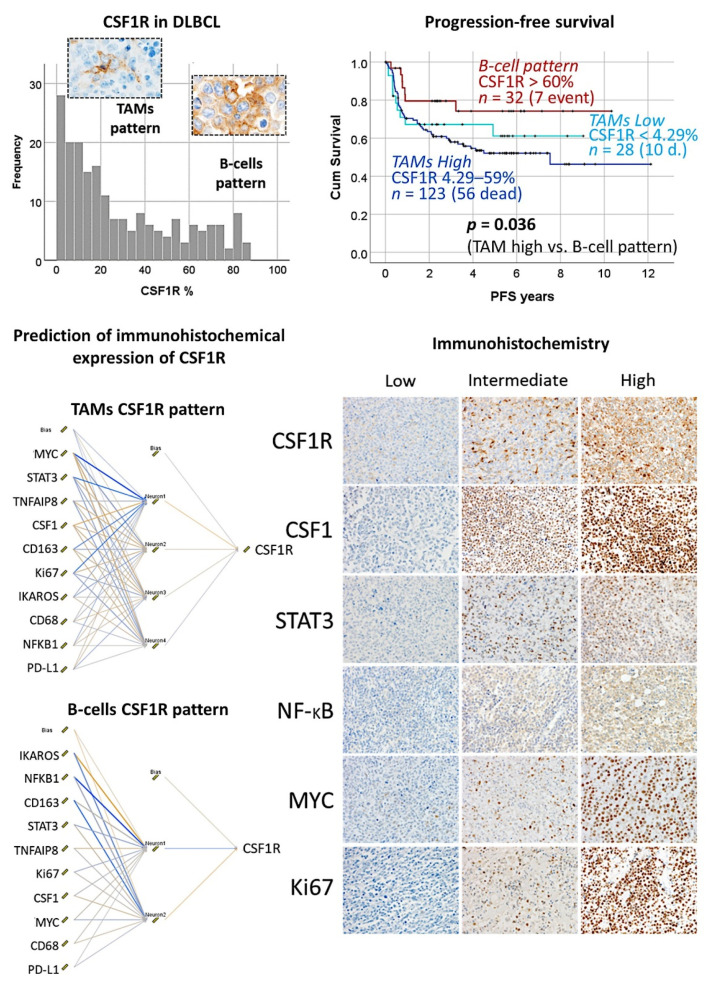
Role of CSF1R in the prognosis of diffuse large B-cell lymphoma. CSF1R was analyzed by immunohistochemistry in a series of 198 cases, and two histological patterns were found. A CSF1R-positive B-cell pattern was characterized by favorable progression-free survival; this pattern was less frequent (around 30% of the cases). Conversely, the most frequent pattern was of CSF1R-positive tumor-associated macrophages (TAMs) and was associated with an unfavorable outcome. Additionally, the prediction of the immunohistochemical expression of CSF1R by other CSF1R-related markers was performed using neural networks. The CSF1R-related markers were CSF1, STAT3, NFKB, MYC, and Ki67. All markers were quantified using digital image analysis. Of note, the multilayer perceptron network analyses were performed to predict both the TAM and the B-cell patterns. Our data suggested that the use of a CSF1R inhibitor such as Pexidartinib could be used in the CSF1R + TAM pattern. CSF1R, macrophage colony-stimulating factor 1 receptor; DLBCL, diffuse large B-cell lymphoma; TAM, tumor-associated macrophage, PFS, progression-free survival.

**Figure 17:**
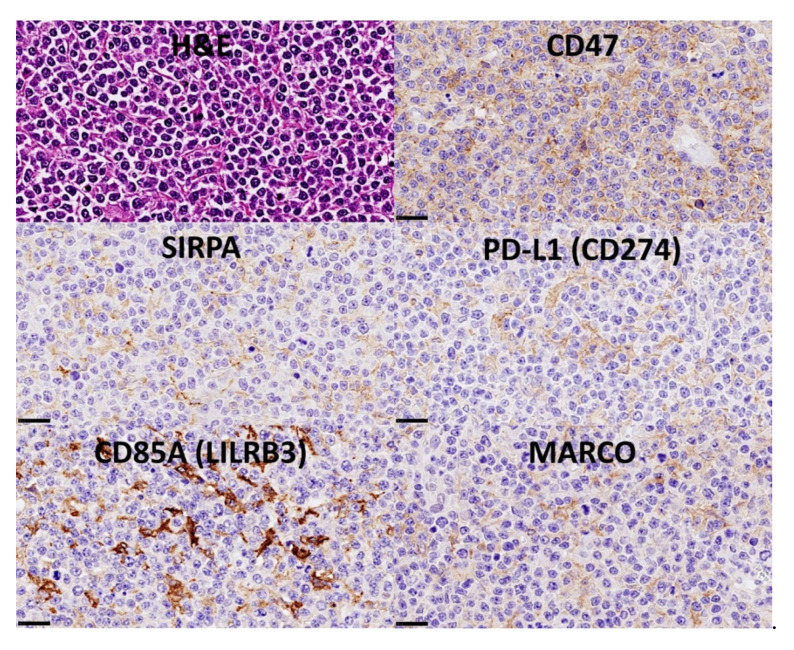
Correlation between expression levels of CSF1R and SIRPA/CD47 in diffuse large B-cell lymphoma. The immunohistochemical pattern of CSF1R-positive tumor-associated macrophages (TAMs) suggested a relationship with other makers such as SIRPA. SIRPA is a relevant immune checkpoint marker that mediates negative regulation of phagocytosis. The histological pattern of SIRPA was of TAMs, similar to PD-L1, CD85A, and MARCO. A ligand for SIRPA is CD47. In our series, the histological pattern of CD47 was of B lymphocytes of the diffuse large B-cell lymphoma.

**Figure 18:**
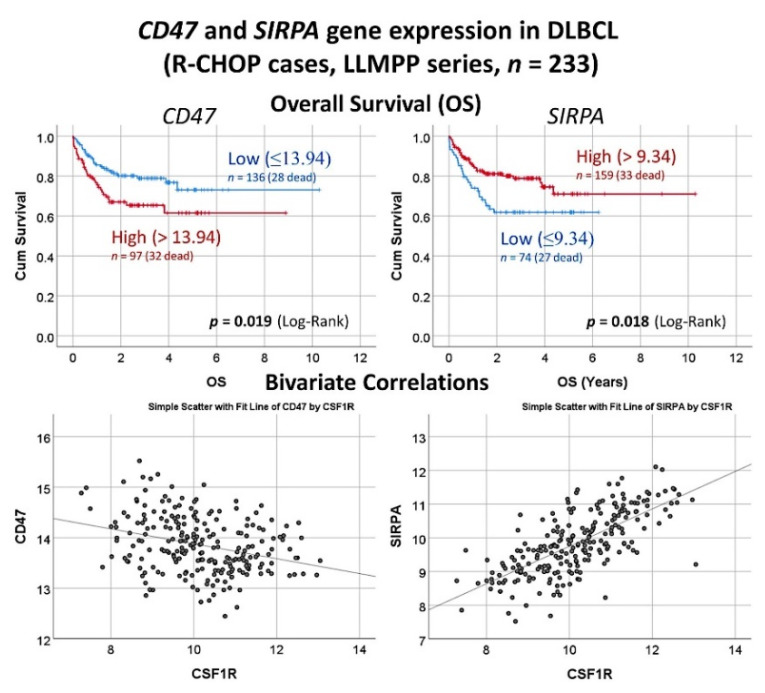
Gene expression analysis of *CD47* and *SIRPA* in the diffuse large B-cell lymphoma. In the series of the Lymphoma/Leukemia Molecular Profiling Project (LLMPP), when analyzing only the cases with R-CHOP-like treatment, high *CD47* but low *SIRPA* correlated with poor overall survival of the patients, and *SIRPA* positively correlated with *CSF1R*. CD47 is a ligand for SIRPA (SIRPα), a protein expressed by macrophages and dendritic cells. These two markers belong to the immune checkpoint pathway, and mediate a negative regulation of phagocytosis. R-CHOP, rituximab, cyclophosphamide, doxorubicin hydrochloride, vincristine, and prednisolone; LLMPP, Lymphoma/Leukemia Molecular Profiling Project; OS, overall survival.

- The protein expression of CSF1R was analyzed by immunohistochemistry in 198 cases of diffuse large B-cell lymphoma, and it was found that high CSF1R-positive TAMs were associated with poor progression-free survival (Figure 16) [32].
- Using a neural network, CSF1R protein expression was predicted by 10 CSF1R-related markers (CSF1, STAT3, NFKB1, Ki67, MYC, PD-L1, TNFAIP8, IKAROS, CD163, and CD68) (Figure 16) [32].
- The gene expression of *CSF1R* was predicted by all the genes, and by an immuno-oncology pattern, and correlated with *SIRPA* and *CD47* [32] (Figure 17 and Figure 18).

### 3.10. Diffuse Large B-Cell Lymphoma, Pan-Cancer Immuno-Oncology Panel

**Figure 19:**
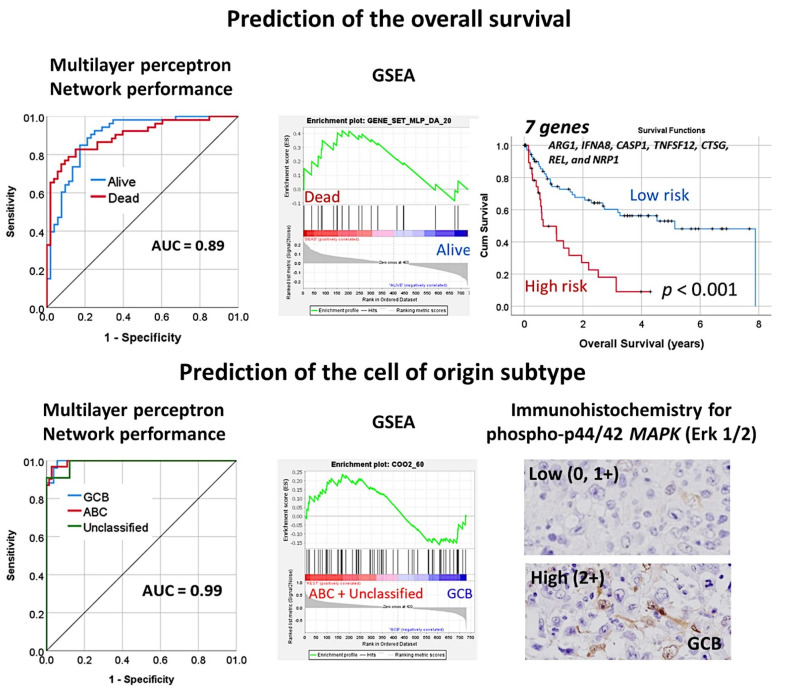
An artificial neural network predicted the overall survival of the diffuse large B-cell lymphoma patients, and the cell of origin subtype using a pan-cancer immuno-oncology gene expression panel. The analysis consisted of the multilayer perceptron. The cell of origin characterization was assessed with the NanoString Lymph2Cx assay. The performance of the network was high, 0.89 for overall survival and 0.99 for the cell of origin phenotype. GSEA analysis confirmed enrichment toward the survival outcome of the dead and the cell of origin subtype of activated (ABC) + unspecified. Using a risk score formula, with 7 genes it was possible to predict the survival of diffuse large B-cell lymphoma. The association of phospho-MAPK with the germinal center B-cell (GCB) phenotype was also noted and confirmed by immunohistochemistry. GSEA, gene set enrichment analysis. ABC, activated B-cell type; GCB, germinal center B-cell type.

- An immuno-oncology panel of 730 genes predicted the overall survival and cell-of-origin phenotype (Lymph2Cx assay) of a series of 106 diffuse large B-cell lymphoma cases, using artificial neural networks and machine learning [33].
- The association of MAPK3 with the GCB phenotype was confirmed by immunohistochemistry [33] (Figure 19).

### 3.11. Diffuse Large B-Cell Lymphoma, Integrative Analysis of Macrophage Markers

**Figure 20:**
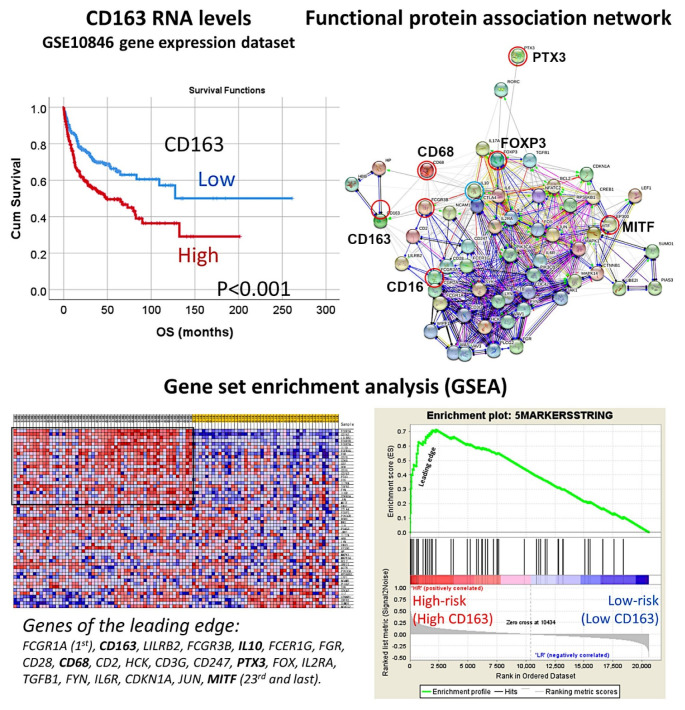
Analysis of macrophages in diffuse large B-cell lymphoma. The overall survival of diffuse large B-cell lymphoma was assessed based on the expression of *CD163*, which is an M2-like macrophage marker. High expression was associated with a poor prognosis of the patients. Then, a protein–protein functional network association analysis was performed using the macrophage markers of CD68 (pan-macrophages), CD16 (M1-like macrophages), CD163 (M2-like), PTX3 (M2c-like), and MITF (M2-like), and the regulatory T lymphocytes (Tregs) marker of FOXP3. The network created a macrophage pathway that was subsequently applied to a gene set enrichment analysis (GSEA). The GSEA confirmed the association of the macrophage pathway with the high-risk group, which was characterized by poor overall survival and high CD163-positive macrophages.

Gene expression profiling of 233 DLBCL patients treated with chemotherapy plus Rituximab was obtained from the series GSE10846, present in the NCBI Gene Expression Omnibus database. The prognostic value for overall survival of the gene expression of *CD163* was first tested and 100 representative cases were selected, which contained high-risk (i.e., high *CD163*) and low-risk cases (i.e., low *CD163*) (Figure 20).

A functional protein association network was created using the five macrophage and one regulatory T lymphocyte (Treg) markers: CD68, CD16, CD163, PTX3, MITF, and FOXP3 as the initial nodes (identifies). Then, the resulting network (i.e., pathway) that contained 57 markers was tested for GSEA analysis in the GSE10846 series of gene expression of diffuse large B-cell lymphoma. We identified the most relevant pathological markers (i.e., genes) that are associated with the prognosis of the patients as follows: high-risk (bad prognosis, and with high *CD163* expression) vs low-risk (good prognosis, low *CD163*). We found that this pathway was enriched in the high-risk phenotype with a NOM p-val < 0.001 and FDR q-val < 0.001. In the enrichment score, we could identify the markers: *CD163* (2nd in the list with a rank metric score of 0.515), *CD16* (FCGR3B, 4th), *CD68* (10th), *PTX3* (15th), and *MITF* (23rd). Of note, *FOXP3* was outside the enrichment set of genes so it was not associated with the high-risk group. Importantly, at fifth position, IL10, was identified. GSEA with markers belonging to the immune regulatory M2c-like TAM pathway was also tested with similar results (Figure 20).

**Figure 21:**
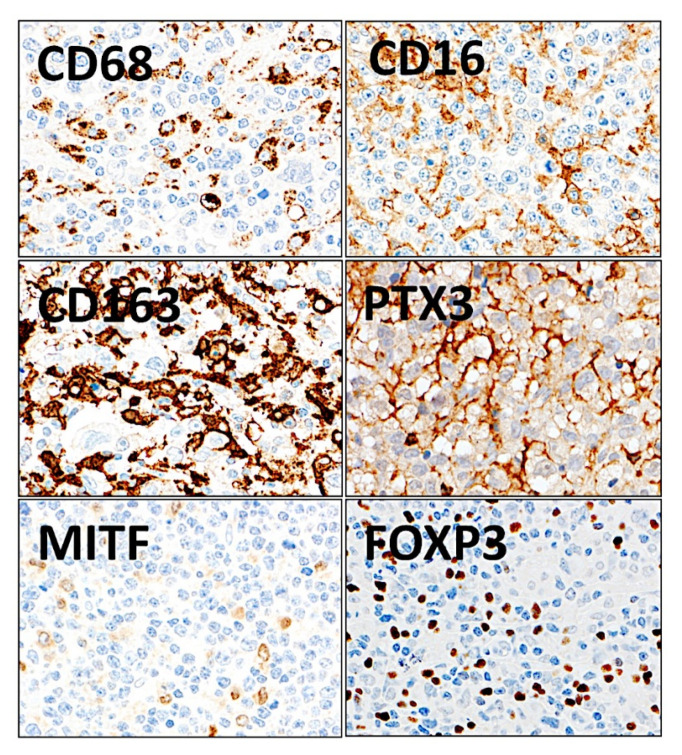
Immunohistochemical staining of macrophage markers and regulatory T lymphocytes (Tregs) in diffuse large B-cell lymphoma. The expression of macrophage markers and Tregs was evaluated using immunohistochemical procedures. The staining confirmed that when macrophages are present at a high concentration in the tissues, their shape is more elongated and dendriform-like. CD68 is a pan-macrophage marker, CD16 is macrophage polarization M1-like, and CD163, PTX3, and MITF are M2-like. FOXP3 is a specific marker of Tregs. Original magnification: 400×.

The macrophage markers were analyzed at protein level by immunohistochemistry in the series of Tokai University (*n* = 132) (Figure 21). The distribution of the markers in the normal reactive tonsil was also evaluated.

The histological analysis in reactive tonsil, a secondary lymphoid organ, showed a different distribution of the different markers. CD68-positive and MITF-positive macrophages were widely distributed in all areas. CD16-positive cells were scarce and only identified in the lympho-epithelium, the epithelial barrier. CD163-positive macrophages were mainly present in the interfollicular regions and infrequently in the germinal centers of the follicles. PTX3-positive cells were of macrophage morphology in all areas and in the germinal centers PTX3-positive cells also had a morphology of B lymphocytes (mainly centroblasts). IL10-positive macrophages were scarce but present in all areas. Double IHC showed mutually exclusive distribution between CD163 and CD16 and partially exclusive with MITF.

**Figure 22:**
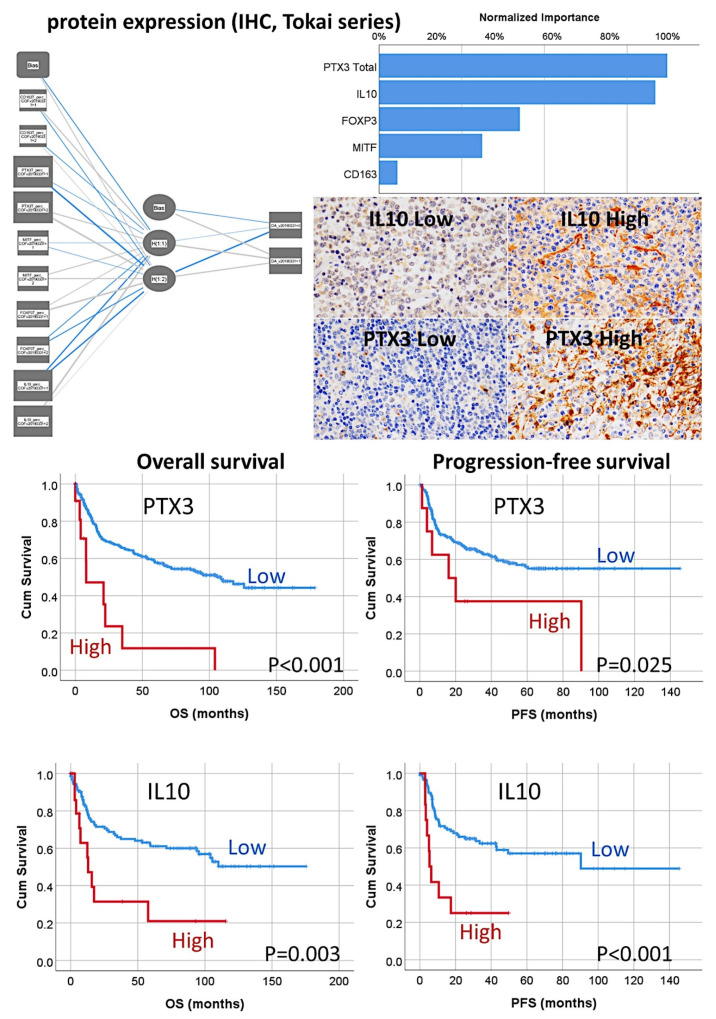
Prediction of the overall survival of diffuse large B-cell lymphoma by M2c-like macrophages using an artificial neural network. The overall survival of the patients was predicted using an artificial neural network using the histochemical data of the tissue samples. The network confirmed that the most relevant markers were PTX3 and IL10, which characterized the immune regulatory M2c-like macrophages. A conventional survival analysis using the Kaplan–Meier with log-rank test confirmed the association of high M2c-like macrophages with poor overall and progression-free survival of the patients. Original magnification: 400×.

The multilayer perceptron (MLP) procedure was performed to produce a predictive model for one target variable, using the values of several predictors. The target was the dead or alive variable for overall survival. The predictors were the same categorical variables used in the COX multivariate analysis: CD163, PTX3 Total, MITF, FOXP3, and IL10. The independent variables normalized importance were as follows: PTX3 Total (100%), IL10 (95.9%), FOXP3 (48.9%), MITF (35.8%), and CD163 (6.3%) (Figure 22). This result is compatible with COX. The same procedure was performed to predict the Hans classifier and the importance was IL10 (100%), PTX3 Total (67.1%), FOXP3 (44.8%), CD163 (39.8%), and MITF (32.8%) (Figure 22).

**Figure 23:**
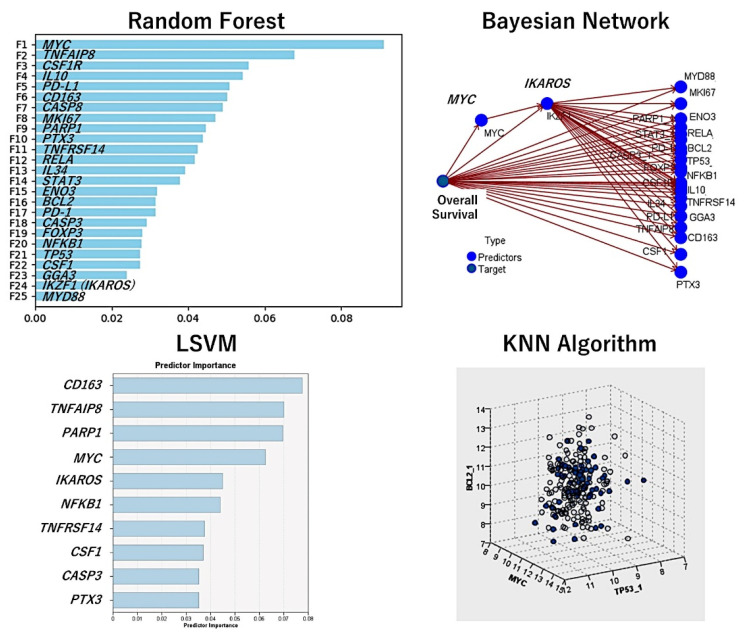
Prediction of the overall survival of diffuse large B-cell lymphoma using immune checkpoint and immuno-oncology markers. Using gene expression data of the GSE10846 dataset, the association of markers of immune regulatory M2c-like tumor-associated macrophages and other immune checkpoint markers was assessed. The methodology included several machine learning and artificial neural networks. The overall accuracy of each method is shown in Table 2.

Additional analysis consisted of validation the macrophage markers in an independent series of cases of diffuse large B-cell lymphoma, from the Lymphoma/Leukemia Molecular Profiling Project (LLMPP), the GSE10846 (webpage: https://www.ncbi.nlm.nih.gov/geo/query/acc.cgi?acc=GSE10846, accessed on 21 September 2022). Only the cases treated with R-CHOP-like therapy were selected (*n* = 233). Several machine learning and artificial neural networks (multilayer perceptron) were used. The dependent (target) variable was the overall survival (outcome dead vs alive). As predictors, the macrophage genes of *CD163, CSF1R, PTX3, CD274 (PD-L1)*, and *IL10* were used. Additional immuno-oncology predictors were markers previously highlighted in the analyses, including *MYC, BCL2, TP53, FOXP3, CSF1, IL34, PDCD1 (PD-1), TNFRSF14, TNFAIP8, IKZF1, STAT3, NFKB1, MYD88, RELA, CASP8, CASP3, PARP1, BCL2, MKI67, ENO3*, and *GGA3*. In total, 25 genes were analyzed and the overall survival was successfully predicted. Table 2 shows the machine learning and neural network models, the number of predictors used in the models, and the overall accuracy. Figure 16 shows the most relevant models and the most relevant genes. The models confirmed the importance of the immuno-oncology markers (Figure 23).

Using the random forest, the markers were ranked according to their significance for predicting the patients’ overall survival. The random forest uses a tree model and a bagging method.

The Bayesian network is a graphical model that shows variables (nodes) in a dataset and the probabilistic, or conditional, independences between them. It constructs a probability model by combining observed and recorded evidence. The network’s links (arcs) do not always depict cause and effect.

The LSVM method permits the classification of data using a linear support vector machine. With large datasets, or ones with numerous predictor fields, LSVM is an especially adequate method. In this LSVM analysis, the predictors were ranked in order of relevance.

Nearest Neighbor Analysis classifies the cases based on the resemblance to others and patterns; this chart is a lower-dimensional projection of the predictor space, which contains 25 predictors (genes).

## 4. Discussion

Artificial intelligence (AI) is a recently developed field that integrates computer science with datasets to perform out calculations. In medicine, both machine learning and deep learning analyze medical data and gain insights on diseases. Artificial intelligence has many applications, including diagnosis, disease classification, image analysis, etc. [20,21,22,23,24].

Machine learning is a specialty in artificial intelligence. By using statistics, algorithms are trained to make classifications or predictions [20,21,22,23]. An algorithm of machine learning is composed of three parts:

(1) Decision process. Based on the labeled or unlabeled input data, an estimated pattern is produced by the algorithm.
(2) Error function, which evaluates the prediction of the model.
(3) Model optimization process. During the fitting, the weights are adjusted to reduce discrepancy between the known and the estimates, and weights are updated autonomously until a threshold of accuracy is met.

There are three categories of machine learning models:

(1) Supervised, which use labeled datasets, such as linear regression, logistic regression, random forest, and support vector machine (SVM).
(2) Unsupervised, which use unlabeled datasets and discover hidden patterns or data groupings without the need of human intervention, such as principal component analysis (PCA), singular value decomposition (SVD), and k-means clustering.

A linear regression algorithm is used to predict numerical values based on a linear relationship between predictors. Logistic regression is a type of supervised learning that predicts a categorical variable (binary). The clustering analysis uses unsupervised learning and identifies patterns to group the cases. Decision trees can be used to predict numerical values or to classify the data into categories; they use a branching sequence of link decisions that are represented in a tree diagram. Random forests predict a value or category by combining the results of decision trees [20].Artificial neural networks (ANNs) are algorithms that, in essence, mimic the human brain. Many data mining applications use neural networks because they are flexible and powerful for complex processes [25].

A neural network is composed of an input layer, multiple hidden layers (deep neural network), and an output layer. Most neural networks are feed-forward, which means that the flow moves in one direction from the input to the output [20,21,22,23,24]. The “deep” term refers to the number of layers (inclusive of input, hidden, and output layer); more than three layers can be considered in a deep learning algorithm [21]. The multilayer perceptron (MLP) and radial basis function (RBF) are used in predictive applications, and are supervised because the results can be compared with the known values of the target variables [20,21,22,23,24,25,26]. The input layer contains the predictors (for example, the genes). The hidden layer contains unobservable nodes (units). The value of each hidden unit is some function of the predictors. The output layer contains the responses (Figure 2).

This research predicted the prognosis (mainly the overall survival) and classified the different subtypes of mature B-cell neoplasms (non-Hodgkin lymphomas) with high accuracy. Therefore, machine learning and artificial neural networks are useful biostatistical tools in biomedical research, and it is expected that the importance of artificial intelligence in medicine will increase in the future.

This research used basic types of neural networks to obtain reliable results. The single neural networks created the basis for more complex algorithms, making the analysis similar to a classical multivariate analysis. The neural networks were also complemented with other conventional biostatistical analyses, such as gene set enrichment analysis (GSEA) and Cox regression. Additionally, other machine learning techniques were used to complement the results. Each type of machine learning has special uses, and in the results, the information that is provided was complementary.

In the different algorithms, the input data comprised all the genes of the array or specific panels. The panels that were used were carefully selected, and included cancer transcriptome, pan-cancer, cancer progression, and metabolic pathways that incorporate many oncogenes and tumor suppressor genes, but also immune-related panels such as immune exhaustion, human inflammation, host response, autoimmune, and immuno-oncology. Nowadays, immuno-oncology panels are particularly relevant. This research highlighted many important immuno-oncology markers such as CD163, CSF1R, CSF1, PD-L1, IL10, TNFRSF14, TNFAIP8, PD-1, and FOXP3 which are markers of tumor-associated macrophages (TAMs), T lymphocytes, and regulatory T lymphocytes (Tregs). A complete discussion can be found in the previous publications [19,27,28,29,30,31,32,33,34,35]. Most of these markers can be targeted using inhibitors. In diffuse large B-cell lymphoma, the use of immunomodulatory drugs and immune checkpoint inhibitors is a new and promising field for treating the patients beyond the classical R-CHOP [58] (Table 3).

Interestingly, some of the identified markers were also relevant for the prognosis of nonhematological neoplasia, which suggests that there are common pathogenic mechanisms in all types of neoplasia.

AI analysis combined neural networks such as multilayer perceptron and radial basis function, and several machine learning techniques such as Bayesian network, C&R tree, C5 tree, CHAID tree, discriminant analysis, KNN algorithm, logistic regression, LSVM, Quest tree, random forest, random trees, SVM, tree-AS, XGBoost linear, XGBoost tree. It is impossible to decide which the best technique is because each method has some strengths and weaknesses, and its applicability depends on the type of data, number of cases, and number of variables (inputs).

The term neural network refers to a family of loosely related models that are characterized by large parameter spaces and flexible structures, derived from the study of brain function. Neural networks are the tools of choice in many data mining applications because of their power and flexibility, especially if the underlying process is complex [28].

Artificial neural networks used in prediction applications, such as multilayer perceptron (MLP) and radial basis function (RBF) networks, are supervised in the sense that the results predicted by the model are compared to known values of target variables. The choice between the MLP and RBF methods depends on the type of data and the level of complexity of the problem. The MLP method can find more complex relationships, while RBF is generally faster [30]. Deep neural networks have been criticized for being opaque because their predictions are incomprehensible to humans; their multi-layered nonlinear structure is a “black box model” [31].

We recently modeled celiac disease and ulcerative colitis using AI [59,60]. In the case of ulcerative colitis, we analyzed a series of 43 cases, including 13 healthy controls, 8 inactive ulcerative colitis, 7 non-involved active ulcerative colitis, and 15 involved active ulcerative colitis. As input, 734 genes were included. A total of 16 models were used to predict ulcerative colitis. The overall accuracy was as follows: C5 decision tree (100%, 2 fields used); logistic regression, discriminant analysis, LSVM, SVM, XGBoost linear, XGBoost tree, and neural network (100%, 734 fields); CHAID (97.7%, 2 fields); random forest (97.7%, 734); KNN algorithm (95.4%, 734); C&R tree (95.4%, 12); Quest (83.7%, 6); Bayesian network (65.1%, 734); random trees (0%, 734). In this research, most of the machine learning methods and neural networks had accuracy above 85%. Nevertheless, the number of fields that were used was variable. As also observed in the data of mature B-cell neoplasms, decision trees have difficulties in handling a large set of variables. Bayesian networks provide acceptable results, but are not superior to neural networks. Logistic regression accuracy is usually high and uses many variables. In the end, the most practical strategy is to test all methods and select the ones that predict better. In Table 2, the same 16 models are applied to our data of diffuse large B-cell lymphoma. Generally, the machine learning methods successfully predicted the overall survival of patients with diffuse large B-cell lymphoma using immuno-oncology and immune checkpoint markers. In this particular experiment, neural networks did not have high accuracy.

In conclusion, artificial intelligence analysis is a useful tool for analyzing the prognosis and classification of non-Hodgkin lymphomas.

## 5. Review of the Literature and Future Perspective in Hematological Neoplasia Using AI

Other groups have also used artificial intelligence in the field of hematopathology research. Table 4 provides precise updates on the latest progress made in hematological malignancies using machine learning and neural networks. The manuscripts were selected in PubMed using the keywords “lymphoma” and “artificial intelligence”. Among all articles that were found within the past 3–4 years, a selection of the most recent research was made. Because of limited space, not all relevant manuscripts are included in Table 4.

The manuscripts were organized according to the type of input data, i.e., PET/CT scan, histological images, immunophenotype, clinicopathological variables, and gene expression, mutational, and integrative analysis-based artificial intelligence [61,62,63,64,65,66,67,68,69,70,71,72,73,74,75,76,77,78,79,80,81,82,83,84].

Worth mentioning is the work of Schmitz R et al. published in the *New England Journal of Medicine* in 2018. The genetics and pathogenesis of diffuse large B-cell lymphoma were analyzed using random forest. The input data from 574 diffuse large B-cell lymphoma cases included exome and transcriptome sequencing, whole-genome copy-number array-based DNA analysis, and targeted amplicon resequencing of 372 genes to identify genetic subtypes [84].

A similar work was published by Xu-Monette ZY et al. in 2020 in *Blood Advances*. Based on targeted next-generation sequencing (NGS), a correlation with the cell of origin subtypes was made using AI in diffuse large B-cell lymphoma. The series of 418 cases included immunohistochemical, gene expression, DNA in situ hybridization, array CGH, and NGS sequencing. Using autoencoders and CPH models, the cases were classified according to the cell of origin and the patients’ survival (overall survival and progression-free survival) [81].

Li D et al. reported in 2020 in *Nature Communications* a deep learning diagnostic platform for diffuse large B-cell lymphoma. The method included data from multiple hospitals. This research used histological images of H&E to classify diffuse large B-cell lymphoma (DLBCL) vs non-DLBCL. Non-DLBCL included cases of metastatic carcinoma, melanoma, and other lymphomas. The lymphoma subtypes were chronic lymphocytic leukemia, mantle cell lymphoma, follicular lymphoma, and classical Hodgkin lymphoma. Seventeen types of convolutional neural networks were used, and the model had an accuracy of 99.7–100% [74].

In the past five years, there has been a significant increase in the use of artificial intelligence in cancer research, and many applications in hematological neoplasia have been published [85]. Many studies have used convolutional neural networks to classify digitalized histological images. Machine learning and artificial neural networks have also been used to analyze gene expression and mutational data. It is expected that in the future, artificial intelligence techniques will become a standard part of the biostatistical analysis, and complementary to “conventional” bioinformatics.

## Figures and Tables

**Table 1:** Immunohistochemical markers used in lymphoma cases of Tokai University, School of Medicine.

Marker	Target/Pathway	Primary Antibody	Company
BCL2	Apoptosis	bcl2/100/D5	Novocastra
BCL6	Germinal center	LN22	Novocastra
cCASP3	Apoptosis	Asp175, #9661	Cell Signaling
CASP8	Apoptosis	active subunit p18, 11B6	Novocastra
CD3	T lymphocytes	CD3 epsilon, LN10	Novocastra
CD5	T lymphocytes	4C7	Novocastra
CD10	Germinal center	56C6	Novocastra
CD16	M1-like macrophages	2H7	Novocastra
CD20	B lymphocytes	L26	Novocastra
CD47	B lymphocytes	D3O7P	Cell Signaling
CD68	Pan-macrophages	514H12	Novocastra
CD85A/LILRB3	M2-like macrophages	FRAS92B	CNIO
CD163	M2-like macrophages	10D6	Novocastra
CDK6	Cell cycle	98D	CNIO
CSF1	CSF1R pathway	2D10	LSBio
CSF1R	M2-like macrophages	2D10	LSBio
Cyclin D1	Cell cycle	P2D11F11	Novocastra
E2F1	Cell cycle	Agro368V	CNIO
EBER	Epstein-Barr virus	#PB0589, #AR0833	Novocastra
IKAROS	Cytokine signaling	D6N9Y	Cell Signaling
IL10	M2c-like macrophages	LS-B7432	Lifespan Bioscience
Ki67	Cell cycle	MM1	Novocastra
LMO2	Proto-oncogene	299B	CNIO
MARCO	Macrophages	HPA063793	Atlas antibodies
MDM2	p53 signaling	IF2	Invitrogen
MITF	M2-like macrophages	C5/D5/MAB10775	Abnova
MUM1	Plasma cells	IRF4, EAU32	Novocastra
MYC	Proto-oncogene	Y69	Abcam
NFKB p105/p50	NFKB pathway	#3035	Cell Signaling
cPARP	Apoptosis	Asp214, D64E10	Cell Signaling
PD-L1	Immune checkpoint	E1J2J	Cell Signaling
p-p44/42 MAPK	MAPK pathway	Thr202/Tyr204, #4370	Cell Signaling
pSTAT3	STAT3 pathway	Tyr705, D3A7	Cell Signaling
PTX3	M2c-like macrophages	PPZ1228	Perseus Proteomics
RGS1	Signal transduction	Rabbit polyclonal	Thermo Fisher
SIRPA	M2-like macrophages	D6I3M	Cell Signaling
TNFAIP8	Apoptosis	#14559-MM01	Sino Biological
TP53	Cell cycle, apoptosis	DO-7	Novocastra
RGS1	Signal transduction	Rabbit polyclonal	Thermo Fisher

CNIO, Centro Nacional de Investigaciones Oncológicas (Spanish National Cancer Research Center).

**Table 2:** Machine learning and artificial neural network analysis using gene expression data.

Model	No. of Predictors	Overall Accuracy (%)
XGBoost Tree	25	100
Random Forest	25	98.3
Random Trees	25	97.1
Bayesian Network	25	89.3
SVM	25	84.5
KNN Algorithm	25	81.9
CHAID	6	79.8
LSVM	25	78.5
Logistic Regression	25	78.1
C5 Tree	3	75.9
Tree-AS	2	74.3
XGBoost Linear	25	74.3
Quest	25	74.3
C&R Tree	25	74.3
Neural Net	25	74.3
Discriminant	25	72.9

**Table 3:** Immuno-oncology and pathway-related markers that were highlighted in this research.

Marker	Target Cell/Pathway	Function/Prognostic Association
FOXP3	Tregs	Immune tolerance and homeostasis of the immune system. High frequency associated with a favorable prognosis of DLBCL.
PD-1	T lymphocytes	Co-inhibition
BTLA	B and T lymphocytes	Co-inhibition
CD163	M2-like TAMs	Pro-tumoral. High frequency is associated with poor prognosis of DLBCL and FL.
CSF1R	M2-like TAMs	Pro-tumoral. High CSF1R + TAMs associated with poor prognosis, but high CSF1R + B-cells of DLBCL with favorable prognosis.
CSF1	B lymphocytes	Ligand of CSF1R
PD-L1	M2c-like TAMs	Pro-tumoral, immune regulatory macrophages (M2c-like). High expression associated with poor prognosis of DLBCL.
SIRPA	M2-like TAMs	Limit phagocytosis
CD47	B lymphocytes	Limit phagocytosis
IL10	M2c-like TAMs	Pro-tumoral, immune regulatory macrophages (M2c-like). High expression associated with poor prognosis of DLBCL and FL.
TNFRSF14	Antigen-presenting cells	Ligand of BTLA, co-inhibitory pathway
IKAROS	Pathway-related	Transcription factor, chromatin remodeling, hemolymphopoietic system. High expression associated with a favorable prognosis of DLBCL.
STAT3	Pathway-related	Cell growth and apoptosis
NFKB1	Pathway-related	Activated by cytokines, oxidant-free radicals, ultraviolet irradiation, and bacterial or viral products. Activated NFKB translocates into the nucleus and stimulates expression multiple genes of wide variety of biological functions.
MAPK	Pathway-related	p44/42 MAPK (Erk1/2) signaling pathway. High expression associated with GCB phenotype of DLBCL (and a favorable prognosis).
TNFAIP8	Pathway-related	Anti-apoptosis. High expression associated with poor prognosis of DLBCL.
BCL2	Pathway-related	Anti-apoptosis
CASP8	Pathway-related	Pro-apoptosis. High expression associated with a favorable prognosis of DLBCL.
CASP3	Pathway-related	Pro-apoptosis
PARP	Pathway-related	Pro-apoptosis
MDM2	Pathway-related	TP53 in inhibitor
E2F1	Pathway-related	Transcription factor, cell cycle, tumor suppressor
CDK6	Pathway-related	Cell cycle
MYB	Germinal center B-cells	Transcriptional transactivator
LMO2	Germinal center B-cells	Hematopoietic development
ENO3	Pathway-related	Glycolysis and glycosaminoglycan metabolism. High expression associated with a poor prognosis of DLBCL.
GGA3	Pathway-related	Positive regulation of protein catabolic processes

Tregs, regulatory T lymphocytes; TAMs, tumor-associated macrophages; DLBCL, diffuse large B-cell lymphoma; FL, follicular lymphoma. Information based on UniProt and GeneCards, and our results.

**Table 4:** Update on the latest progress made in hematological malignancies using artificial intelligence.

Authors (Year)	Journal	Research Title	Summary	Technique Used	Reference
*(1) PET/CT scan-based AI*					
Lisson CS et al. (2022)	*Cancers (Basel)*	Deep neural networks and machine learning radiomics modeling for the prediction of relapse in mantle cell lymphoma	This research predicted the relapse of mantle cell lymphoma (MCL) using baseline CT scans. The accuracies of predictions ranged from 64% to 70%.	3D SEResNet50, 3D DenseNet, optimized 3D CNN, K-nearest Neighbor (KNN), and Random Forest (RF)	[61]
Sadik M et al. (2021)	*Sci Rep.*	Artificial intelligence could alert for focal skeleton/bone marrow uptake in patients with Hodgkin’s lymphoma staged with FDG-PET/CT	Detection of focal skeleton/bone marrow uptake (BMU) in patients with Hodgkin’s lymphoma (HL) undergoing staging with FDG-PET/CT. Training set, *n* = 153; validation set, *n* = 48.	Convolutional neural network (CNN)	[62]
Wang YJ et al. (2021)	*Eur J Nucl Med Mol Imaging*	Artificial intelligence enables whole-body positron emission tomography scans with minimal radiation exposure	Thirty-three diagnostic ^18^F-FDG PET images of patients with pediatric cancer were generated from ultra-low dose ^18^F-FDG PET input images using an AI algorithm. Then, the AI-generated PET scans were compared with clinical standard PET scans.	Convolutional neural network (CNN)	[63]
Pinochet P et al. (2021)	*Front Med (Lausanne)*	Evaluation of an automatic classification algorithm using convolutional neural networks in oncological positron emission tomography	This research measured the efficiency and performance in both clinical and research environments of a system called positron emission tomography (PET)-assisted reporting system (PARS) (Siemens Healthineers). The method was based on a convolutional neural network (CNN) that identified suspected cancer sites in fluorine-18 fluorodeoxyglucose (18F-FDG) PET/computed tomography. These data were correlated with the survival of the patients. Two cohorts were evaluated: 119 cases of DLBCL, and 430 cases of DLBCL and other tumors.	Dice score	[64]
*(2) Histological images-based AI*					
El Hussein S et al. (2022)	*J Pathol.*	Artificial intelligence strategy integrating morphologic and architectural biomarkers provide robust diagnostic accuracy for disease progression in chronic lymphocytic leukemia	Cytologic and architectural features obtained from whole-slides images were used to classify 125 samples into three subtypes: chronic lymphocytic leukemia (CLL, *n* = 69), progression to accelerated CLL (aCLL, *n* = 44), and transformation to diffuse large B-cell lymphoma (Richter transformation; RT, *n* = 80).	Hover-Net	[65]
Swiderska-Chadaj Z et al. (2021)	*Virchows Arch.*	Artificial intelligence to detect *MYC* translocation in slides of diffuse large B-cell lymphoma	The H&E slides of 287 cases were evaluated using a deep learning algorithm to identify *MYC* rearrangement by DNA in situ hybridization (FISH).	Deep learning neural network (U-Net) and classical machine learning (random forest classification)	[66]
Steinbuss G et al. (2021)	*Cancers (Basel)*	Deep learning for the classification of non-Hodgkin lymphoma on histopathological images	In this research, the training set included 84,139 image patches from 629 patients that were classified as reactive lymph nodes, nodal small lymphocytic lymphoma/chronic lymphocytic leukemia, and nodal diffuse large B-cell lymphoma. The validation set included 16,960 image patches from 125 patients. The final model had an accuracy of 96%.	EfficientNet convolutional neuronal network (CNN)	[67]
Zhang X et al. (2021)	*Technol Health Care*	Research on the classification of lymphoma pathological images-based on deep residual neural networks	The analysis used 374 pathological images, including chronic lymphocytic leukemia, follicular lymphoma, and mantle cell lymphoma.	BP neural network and BP neural network optimized by genetic algorithm (GA-BP), deep residual neural network model (ResNet50), softmax layer	[68]
Tang G et al. (2021)	*Acta Cytol.*	A machine learning tool using digital microscopy (Morphogo) for the identification of abnormal lymphocytes in the bone marrow	Morphological differentiation of abnormal lymphocytes in bone marrow was evaluated in 53 cases of different subtypes of B-cell lymphomas, using automated digital images.	“Morphogo” system	[69]
Yu WH et al. (2021)	*Cancers (Basel)*	Machine learning based on morphological features enables the classification of primary intestinal T-cell lymphomas.	A total of 40 primary intestinal T-cell lymphomas (PITL), including 26 monomorphic epitheliotropic intestinal T-cell lymphoma (MEITL), 10 intestinal T-cell lymphoma, not otherwise specified (ITCL-NOS), and 4 borderline cases were analyzed. The inputs were the morphological features and the immunophenotypes (CD8 and CD56).	XGBoost and CNN (HTC-RCNN with ResNet50)	[70]
Zhou M et al. (2021)	*Front Pediatr.*	Development and evaluation of a leukemia diagnosis system using deep learning in real clinical scenarios	A total of 1732 bone marrow, raw images of 89 children with leukemia were analyzed with convolutional neural networks, with a performance accuracy of 89%. Apart from detecting leukocytes, the system also detected bone marrow metastasis of lymphoma and neuroblastomas.	RetinaNet, VGG, Feature Pyramid Network, ResNet, convolutional neural network (CNN)	[71]
Zhang J et al. (2020)	*Med Phys.*	Classification of digital pathological images of non-Hodgkin’s lymphoma subtypes based on the fusion of transfer learning and principal component analysis	Digital pathology images of non-Hodgkin lymphoma, including chronic lymphocytic leukemia (CLL), follicular lymphoma (FL), and mantle cell lymphoma (MCL) tumor were analyzed and classified. The model had an overall accuracy of 98.9%.	Transfer learning (TL) and principal component analysis (PCA)	[72]
Mohlman JS et al. (2020)	*Am J Clin Pathol.*	Improving augmented human intelligence to distinguish Burkitt lymphoma from diffuse large B-cell lymphoma cases	A total of 10,818 H&E images from 34 cases of Burkitt lymphoma and 36 cases of diffuse large B-cell lymphoma were used to train and differentiate the two lymphoma subtypes.	Convolutional neural network (CNN)	[73]
Li D et al. (2020)	*Nat Commun.*	A deep learning diagnostic platform for diffuse large B-cell lymphoma with high accuracy across multiple hospitals	This research used histological images of H&E to classify diffuse large B-cell lymphoma (DLBCL) vs non-DLBCL. Non-DLBCL included metastatic carcinoma, melanoma, and other lymphomas including small lymphocytic lymphoma/chronic lymphocytic leukemia, mantle cell lymphoma, follicular lymphoma, and classical Hodgkin lymphoma. The GOTDP-MP-CNNs (with combined 17 CNNs) model had an accuracy of 99.7% to 100%.	17 types of CNN: AlexNet, GoogLeNet (ImageNet), GoogLeNet (Places365), ResNet18, ResNet50, ResNet101, Vgg16, Vgg19, Inceptionv3, InceptionResNetv2, SqueezeNet, DenseNet201, MobileNetv2, ShuffleNet, Xception, NasNetmobile, Nasnetlarge	[74]
Miyoshi H et al. (2020)	*Lab Invest.*	Deep learning shows the capability of high-level computer-aided diagnosis of malignant lymphoma.	The H&E images of 388 cases, including 259 with diffuse large B-cell lymphoma, 89 with follicular lymphoma, and 40 with reactive lymphoid hyperplasia, were analyzed using deep learning. The accuracy of the model was 97%.	Convolutional neural network (CNN)	[75]
Zorman M et al. (2011)	*Wien Klin Wochenschr.*	Classification of follicular lymphoma images: a holistic approach with symbol-based machine learning methods.	Analysis of follicular lymphoma images, focusing on the identification of follicles.	Decision trees (MtDeciT 3.1, RSES 2.2, and Weka 3) and artificial neural networks (multilayer perceptron)	[76]
*(3) Immunophenotype-based AI*					
Zhao M et al. (2020)	*Cytometry A.*	Hematologist-level classification of mature B-cell neoplasms using deep learning on multiparameter flow cytometry data	Information captured by multiparameter flow cytometry (MFC) of 18,274 cases, including chronic lymphocytic leukemia and its precursor monoclonal B-cell lymphocytosis, marginal zone lymphoma, mantle cell lymphoma, prolymphocytic leukemia, follicular lymphoma, hairy cell leukemia, lymphoplasmacytic lymphoma were analyzed; the model was tested on a set of 2346 cases. The model performance had an F1 score of 0.94.	Self-organizing maps and convolutional neural networks	[77]
Gaidano V et al. (2020)	*Cancers (Basel)*	A clinically applicable approach to the classification of B-cell non-Hodgkin lymphomas with flow cytometry and machine learning	The immunophenotype data from flow cytometry of 1465 B-cell non-Hodgkin lymphoma (NHL) cases were analyzed. The cases included chronic lymphocytic leukemia (CLL), diffuse large B-cell lymphoma (DLBCL), Burkitt lymphoma (BL), follicular cell lymphoma (FCL), hairy cell leukemia (HCL), splenic lymphoma (SL), mantle cell lymphoma (MCL), marginal zone lymphoma (MZL), and lymphoplasmacytic lymphoma (LPL). The accuracy of the classification ranged from 92% to 100%.	Classification trees	[78]
*(4) Clinicopathological variables-based AI*					
Zhan M et al. (2021)	*Leuk Lymphoma*	Machine learning to predict high-dose methotrexate-related neutropenia and fever in children with B-cell acute lymphoblastic leukemia	A model included 57 SNPs of 16 genes and clinical variables to predict neutropenia and fever in 139 pediatric cases of acute lymphoblastic leukemia treated with high-dose methotrexate (MTX).	Random forest	[79]
Buciński A et al. (2010)	*Eur J Cancer Prev.*	Contribution of artificial intelligence to the knowledge of prognostic factors in Hodgkin’s lymphoma	A total of 31 variables from 114 patients with Hodgkin’s lymphoma were used to predict the prognosis of the patients.	Artificial neural network (ANN)	[80]
*(5) Gene expression, mutational, and integrative analysis-based AI*					
Carreras J et al. (2022)	*Healthcare (Basel)*	Artificial intelligence analysis of gene expression predicted the overall survival of mantle cell lymphoma and a large pan-cancer Series	The gene expression data of 123 cases of mantle cell lymphoma (MCL) were analyzed with artificial neural networks to predict the overall survival of the patients with high accuracy. The survival of diffuse large B-cell lymphoma (DLBCL), and a pan-cancer series was also predicted.	Several machine learning techniques, and artificial neural networks	[34]
Carreras J et al. (2021)	*Cancers (Basel)*	Artificial neural networks predicted the overall survival and molecular subtypes of diffuse large B-cell lymphoma using a pan-cancer immune-oncology panel	The gene expression of an immuno-oncology panel of a series of 106 cases of diffuse large B-cell lymphoma was analyzed using artificial intelligence to predict the overall survival and the cell of origin molecular subtypes. The model had a high accuracy of classification.	Several machine learning techniques, and artificial neural networks	[33]
Carreras J et al. (2021)	*Tokai J Exp Clin Med.*	Artificial intelligence analysis of gene expression data predicted the prognosis of patients with diffuse large B-cell lymphoma	The gene expression of a series of 414 cases of diffuse large B-cell lymphoma (DLBCL) was analyzed to predict the overall survival, and was correlated with other known pathogenic genes such as *BCL2* and *MYC.*	Artificial neural networks (ANN)	[27]
Xu-Monette ZY et al. (2020)	*Blood Adv.*	A refined cell of origin classifier with targeted NGS and artificial intelligence showed robust predictive value in DLBCL	The series of diffuse large B-cell lymphoma of 418 cases included immunohistochemical, gene expression, DNA in situ hybridization, array CGH, and NGS sequencing. Using an autoencoder, the cases were classified according to the cell of origin and the survival (overall survival and progression-free survival).	Autoencoder, logistic regression, and CPH models	[81]
Zhang W et al. (2020)	*BMC Cancer*	Novel bioinformatic classification system for genetic signature identification in diffuse large B-cell lymphoma	A total of 342 cases of diffuse large B-cell lymphoma were analyzed using mutational data from a panel of 46 genes by NGS.	Random forest	[82]
Parodi S et al. (2018)	*Health Informatics J.*	Logic learning machine and standard supervised methods for Hodgkin’s lymphoma prognosis using gene expression data and clinical variables	The data of 130 patients diagnosed with Hodgkin’s lymphoma, including a small set of clinical variables and more than 54,000 gene features, were used to predict the prognosis.	K-nearest neighbor (KNN), artificial neural network (ANN), support vector machine (SVM), decision tree, and the innovative logic learning machine method	[83]
Schmitz R et al. (2018)	*N Engl J Med.*	Genetics and pathogenesis of diffuse large B-cell lymphoma	The data of 574 diffuse large B-cell lymphoma cases, which included exome and transcriptome sequencing, array-based DNA copy-number analysis, and targeted amplicon resequencing of 372 genes, were used to identify genetic subtypes.	Random forest	[84]

H&E, hematoxylin and eosin. The publications were selected from PubMed using the keywords “artificial intelligence” and “lymphoma”.

## Data Availability

All the data are available upon request to Joaquim Carreras.

## Supplementary Materials

The following supporting information can be downloaded at: https://www.mdpi.com/article/10.3390/cancers14215318/s1, Table S1: Multilayer perceptron analysis (MLP). Table S2: Radial basis function analysis (RBF). Table S3: Genes associated to poor prognosis in the multivariate Cox survival analysis. Table S4: Genes associated to good prognosis in the multivariate Cox survival analysis. Table S5: Clinicopathological correlations with the final set of seven prognostic genes.

## Appendix A

The analyses used several software applications, including EditPad Lite (version 8.4.0 x64, Just Great Software Co. Ltd.); Fiji (version ImageJ 1.53u, NIH); GSEA (version 4.3.2, Broad Institute); GIMP (version 2.10.8, GNU); IBM SPSS 25 to 27; IBM modeler 18 (IBM); JMP Pro 14 (JMP Statistical Discovery LLC, SAS); Microsoft excel 2016 (version 16.0.5317.1000, Microsoft Corporation); Minitab (version 21.1.0, Minitab, LLC); Morpheus matrix visualization and analysis software (version 1, https://github.com/cmap/morpheus.js, Broad Institute) (accessed date 25 October 2022); NSolver (version 4.0, NanoString); RapidMiner Studio (version 9.10.011, RapidMiner); R (version 4.2.1) (http://cran.r-project.org) (accessed date 25 October 2022); RStudio (version 2022.07.2, Build 576, RStudio, PBC); STRING protein–protein interaction networks (version 11.5, STRING Consortium 2022); and Xlstat (Premium 2018.1, Build 49320 x64, multilingual, Addinsoft).

## Appendix B

**Table A1:** Publicly available datasets used in addition to the Tokai University series.

Diagnosis	Dataset	No. of Cases	Reference
Non-Hodgkin lymphomas	GSE132929	290	[40]
Follicular lymphoma		65	
Mantle cell lymphoma		43	
Diffuse large B-cell lymphoma		100	
Burkitt lymphoma		59	
Marginal zone lymphoma		23	
Chronic lymphocytic leukemia	GSE22762	107	[41,42]
	ICGC CLLE-ES	201	
Diffuse large B-cell lymphoma	GSE10846	414	[43,44]
	GSE23501	69	[45]
	GSE4475	159	[46,47]
	TCGA-DLBCL v.2016	47	
	E-TABM-346	52	[48]
Follicular lymphoma	GSE16131	180	[49]
Mantle cell lymphoma	LLMPP Rosenwald 2003	92	[50]
	GSE93291	123	[51]
Multiple myeloma	GSE2658	559	[52,53,54,55,56,57]
Acute Myeloid Leukemia	TCGA-AML v.2016	149	

## Appendix C. Comments and Analysis Of breast Cancer Detection Using Deep Neural Networks

Breast cancer is the second most frequent type of cancer in women, just before skin cancer. Worldwide, breast cancer represents the 30% of all female cancers, and it has a mortality of about 15%, but in emergent countries can reach up to 70% [86,87]. The worldwide incidence ranges from 27 to 97 cases for 100,000 [87], and in about 10% of the cases, there is a genetic predisposition or family history [87]. The most frequently associated germline mutations affect the *BRCA1* and *BRCA2* genes [88,89].

The development of strategies for the early detection of breast cancer is necessary to improve access to treatment and reduce the mortality rate. As described by Basurto-Hurtado JA et al. [90], breast cancer detection includes four steps: image acquisition, segmentation and pre-processing, feature extraction, and classification [90].

Image acquisition can be obtained through several methods, such as mammography, ultrasound, magnetic resonance imaging (MRI), and other approaches, including microwave, computed tomography (TC), and positron emission tomography (PET) [90].

The image processing and classification strategies include several steps: region of interest (ROI) estimation, and feature extraction. The classifiers can be both unsupervised and supervised. Examples of unsupervised classifiers include K-means and hierarchical clustering. Examples of supervised classifiers are decision trees, random forests, AdaBoost, support vector machines, artificial neural networks, and convolutional neural networks [90]. Recently, new image generation techniques have developed, such as infrared thermography (IRT). This technique has been successfully applied to breast cancer; the classification methods included several machine learning and artificial neural networks, and the accuracy ranged from 90% to 100% [90,91,92,93,94,95,96,97,98,99,100,101].

Recently, new classification algorithms have been developed, including autoencoders, deep belief networks, ladder networks, and deep neural network (DNN)-based algorithms such as the deep Kronecker neural network [90,102].

Gene expression profiling is a useful tool in medical research, both for diagnosis and for the elucidation of the disease pathogenesis. Artificial neural networks can handle gene expression profiling data successfully, and we recently described their usability in hematological neoplasia [27,28,29,30,31,32,33,34,35]. In our research, we used conventional machine learning techniques and artificial neural networks because the aim was to identify prognostic factors in a reliable and systematic manner instead of developing new advanced mathematical algorithms. Nevertheless, the performance of the artificial neural networks can be improved with the use of adaptive activation functions (AAFs). Kronecker neural networks (KNNs) are a new type of neural network with adaptive activation functions described by Jagtap AD et al. [103]. Unlike the traditional neural network architecture, in a KNN, the output of the neuron passes to more than one activation function [103]. The use of the Kronecker product in the KNN made the network wide, while at the same time, the number of trainable parameters remained low [103]. Recently, a multi-level KNN approach was used in the analysis of MRI images of brain tumors (glioma) to develop an automated glioma segmentation system [104].

The research in this manuscript focuses on immuno-oncology markers, as we have recently described [85]. In relation to breast cancer, we tested the prognostic value of a set of 718 genes from a pan-cancer immune profiling panel on the overall survival of the patients. A series of 1215 breast cancer patients from The Cancer Genome Atlas (TCGA) was selected. Unfortunately, in this model, a multilayer perceptron analysis failed to properly predict the overall survival of the patients (83.7% overall percent of correct classification, AUC = 0.61). Next, the input was narrowed to 16 genes: macrophage markers (*CD68*, *CSF1R*, *CD163*, *CSF1R*, *CSF1*, *IL10*, *CD274 (PD-L1)*, and *TNFAIP8*), T helper cells (*PDCD1/PD-1*), Tregs (*FOXP3*), apoptosis (*BCL2*, *CASP3*, and *CASP8*), NFKB pathway (*STAT3*), and metabolism (*ENO3*, *GGA3*). The overall survival of breast cancer was predicted using 16 models, namely C5, logistic regression, Bayesian network, discriminant analysis, KNN algorithm, LSVM, random trees, SVM, tree-AS, XGBoost linear, XGBoost tree, CHAID, Quest, C&R tree, random forest, and neural network (multilayer perceptron). Among all models, only random forest provided suitable modeling (input = 16 fields, overall accuracy 98.4%). The order of predictor importance was *CD274*, *FOXP3*, *ENO3*, *IL10*, *CSF1R*, *CSF1*, *BCL2*, *GGA3*, *TNFAIP8*, *CASP8*, *PDCD1*, *CASP3*, *CD163*, *TNFRSF14*, *CD68*, and *STAT3*.

Noteworthy, further analysis was performed in the breast series of the TCGA and the pan-cancer immune profiling panel. In addition to the overall survival, other survival variables were tested, including the disease-specific survival, disease-free interval, and progression-free interval. The multilayer perceptron analysis also failed to predict the survival of the patients with good performance. Additional analyses were performed. Different types of training were tested: batch, online, and mini-batch. Two types of optimization algorithms were also tested: scaled conjugate gradient, and gradient descent. The training options for the scaled conjugate gradient were the following: initial lambda (0.0000005), initial sigma (0.00005), interval center (0), and interval offset (±0.5). The training options for the gradient descent were initial learning rate (0.4), momentum (0.9), interval center (0), and the interval offset (±0.5). Of note, batch training can use both a scaled conjugate gradient and gradient descent. However, online and mini-batch are restricted to gradient descent. The training options of gradient descent in case of online and mini-batch were initial learning rate (0.4), lower boundary of learning rate (0.001), learning rate reduction, in epochs (10), momentum (0.9), interval center (0), and interval offset (±0.5). We tried improving the network performance by changing all the training parameters, but no significant improvement in performance was achieved.
